# Psychosocial responses to outdoor artificial light at night (ALAN): a scoping review

**DOI:** 10.3389/fpsyg.2026.1797689

**Published:** 2026-05-29

**Authors:** Kévin Nadarajah, Chloé Gryczynski-Costa, Mehdi Chahir, Tivizio Pavic, Alain Somat, Stéphanie Bordel

**Affiliations:** 1 Équipe Recherche Psychologie Appliquée (PsyCAP), Centre d'Études et d'Expertise sur les Risques, l'Environnement, la Mobilité et l'Aménagement (Cerema), Saint-Brieuc, France; 2 Department of Psychology, Laboratoire de Psychologie: Cognition, Comportement, Communication (LP3C), EA 1285, Université Rennes 2, Rennes, France

**Keywords:** artificial light at night (ALAN), environmental psychology, psychosocial responses, public lighting, scoping review

## Abstract

**Introduction:**

Artificial light at night (ALAN) has sharply increased due to urbanization and expanded nighttime activities. Given its ecological, health, and energy impacts, several countries and cities have begun implementing lighting reductions. Understanding the psychosocial processes and determinants shaping individuals' responses to ALAN is essential for policy acceptance and effectiveness. The objective of this review is to map and synthesize the scientific literature on psychosocial processes and determinants underlying responses to outdoor ALAN, drawing on concepts from environmental psychology.

**Method:**

Following PRISMA-ScR guidelines, 75 (*N* = 75) studies from seven databases were analyzed to examine psychosocial responses associated with ALAN. Included articles met the following criteria: (1) the article should examine psychosocial responses to outdoor ALAN, such as perceptions, emotions, attitudes, or behaviors, as captured by a wide range of terms used in the literature, (2) present an empirical design, (3) be written in English. This review examines (1) methodological approaches, (2) main psychosocial dimensions, and (3) reported findings.

**Results:**

Four main findings emerged from this review: (1) ALAN research is recent and concentrated in a few countries, (2) studies mainly use quantitative and cross-sectional designs, but varied measures limit comparability, (3) perceptual and emotional responses dominate the literature, while cognitive, motivational, and behavioral dimensions are less studied, and (4) theoretical frameworks, particularly in environmental psychology, are rarely used.

**Discussion:**

Research on psychosocial responses associated with ALAN is dominated by perceptual and emotional approaches, while key psychosocial processes and determinants (e.g., norms, values, and constraints), central to environmental psychology frameworks, remain underexplored. Future work should adopt environmental psychology models to examine how beliefs, emotions, and routines shape individuals' responses to ALAN and inform acceptable lighting policies.

## 1 Introduction

Artificial light at night (ALAN) refers to any type of lighting (e.g., urban, industrial, road lighting, etc.) that reduces natural darkness (Gursky and Grow, 2024). Outdoor ALAN has enabled human activities to extend beyond the day/night cycle and has gradually spread throughout the world (Falchi et al., 2016; Fouquet and Pearson, 2006; Liu et al., 2023; Wang et al., 2023). In recent decades, ALAN has increased significantly (Zapata et al., 2019), particularly in urban areas, where artificial sky brightness has increased by approximately 9.6% per year between 2011 and 2022 (Kyba et al., 2023). This trend is part of a global urbanization process, as the United Nations predicts that the proportion of the population living in urban areas will increase from 56% in 2021 to 68% in 2050 (UN-Habitat, 2022). It has already been estimated that excessive artificial light affects at least 80% of the world's population and over 99% of Europeans (The Lancet Regional Health-Europe, 2023).

Outdoor ALAN contributes to global economic growth, particularly by promoting human mobility and professional and recreational activities (Fotios et al., 2024; Vidal-Tortosa and Lovelace, 2024; Wang et al., 2023). Yet these benefits also involve significant costs, as a city's energy expenditure can account for up to 70% of total consumption (Mills, 2002; Verlichting, 2008) and reach several billion dollars per year (U.S. DOE, 2013). Light pollution is considered a negative consequence of the use of green spaces and outdoor recreational areas (ALAN) (Narisada and Schreuder, 2013), which has harmful effects on the environment and humans (Hao et al., 2024). The International Dark-Sky Association (IDA) (2023) distinguishes between different forms of light pollution, such as sky brightness, glare, light intrusion, and light clutter. The International Commission on Illumination (CIE) defines light pollution as the cumulative adverse effects of artificial lighting. It also disrupts ecosystems and threatens biodiversity by altering the life cycles of animals and plants (e.g., Candolin, 2024; Hölker et al., 2021; Longcore, 2024). This pollution, whether indoor or outdoor, also affects human health as it has a direct impact on sleep, metabolism (e.g., risk of obesity, cardiovascular disease, etc.), mental health, and the occurrence of chronic diseases such as hypertension, diabetes, and cancers (e.g., Cao et al., 2023; Jiménez et al., 2025; Xu et al., 2023). Indeed, nighttime exposure to ALAN leads to suppression of melatonin secretion and disrupts: circadian rhythms and sleep regulation, metabolic and hormonal functions, and immune function (Lei et al., 2024; Walker et al., 2022).

Faced with these multiple challenges and likely motivated by rising energy costs (Zhang et al., 2023, 2024), in several countries policies have been developed to reduce ALAN in order to lower associated costs and lessen impacts on biodiversity and health (Australian Government, Department of Climate Change, 2023; DARKERSKY4CE, 2024; Department for Levelling Up, 2019). Such initiatives appear to be a relevant solution, particularly for reducing CO_2_ emissions linked to energy consumption, but also for limiting harmful effects on ecosystems (Hölker et al., 2021; Calvin et al., 2023; Saad et al., 2021). However, their implementation is not solely a technical consideration; it also involves social changes (e.g., practices, norms, social representations, etc.). Despite the fact that ALAN is likely to have an impact on individuals, both through its direct effects and the measures taken to reduce it, most studies have focused primarily on technical, environmental or health aspects, leaving aside psychological and social aspects (Bordel et al., 2025; Challéat et al., 2018; Rodrigo-Comino et al., 2023; Svechkina et al., 2020a; Wang et al., 2023). Nevertheless, a few reviews have broadened their scope of investigation to go beyond biological, ecological, or health aspects. For example, Wang et al. (2023) outline the positive effects of ALAN (e.g., increased outdoor activities, sense of security) and contrast them with the negative effects (e.g., increased suicidal behavior, moving house, decreased sense of belonging to a place). Some studies also highlight social inequalities related to lighting and its impact on beliefs, such as the association of brightness with safety or prosperity (Zielinska-Dabkowska et al., 2023; Gaston and Miguel, 2022), or the sectors concerned, such as ecology, health, wellbeing, tourism and public safety (Balafoutis et al., 2025), and show that expertise is accompanied by growing concern about light pollution. However, these subjective and social dimensions, which may influence the perception of risks associated with ALAN, are not the primary focus of the studies (Menculini et al., 2024). Yet, taking into account individuals' psychosocial reactions to artificial lighting at night could help predict the extent to which they are likely to accept policies aimed at reducing it (e.g., Boomsma and Steg, 2014b). Thus, outdoor ALAN, considered as an integrated socio-ecological phenomenon, requires consideration of technical, biological, and ecological aspects, as well as social, cultural, psychological, and political aspects (Challéat et al., 2015, 2021). This type of approach refers to the field of environmental psychology (Gifford, 2007; Proshansky et al., 1970), whose main objective is to analyze the interactions between individuals and their physical and social environments. For instance, three models are particularly prevalent in the literature to explain environmental behavior and the acceptance of environmental policies (e.g., Steg and Vlek, 2009; Klöckner, 2013): the Norm Activation Model (NAM, Schwartz, 1977), the Value–Belief–Norm model (VBN, Stern, 2000) and the Comprehensive Action Determination Model (CADM, Klöckner and Blöbaum, 2010). Indeed, taken together these models provide a useful interpretative basis for organizing selected psychosocial dimensions addressed in the literature, such as cognitions, emotions and affective reactions, assessment of consequences and risk perception, personal motivations, norms and values, perceived or organizational constraints, and observed behaviors and uses. In the case of outdoor ALAN, using such an approach could be relevant for understanding how people perceive and interpret their lighting environment (Boyce, 2014; Veitch and Newsham, 2000), and are likely to accept change.

To the authors' knowledge, no literature review has sought to assess psychosocial responses, which remain fragmented, not only in urban settings but also across various spatial contexts (e.g., peri-urban, or rural). This article aims to address this gap by conducting an exploratory review of the existing literature, following the PRISMA-ScR methodology. The objective of this study is to map and synthesize the scientific literature on psychosocial responses to outdoor ALAN, with a particular focus on the psychosocial processes and determinants addressed in this body of work. More specifically, the review examines (1) the methodological approaches used to study these responses, (2) the main dimensions and concepts that have been studied, and (3) the results about psychosocial aspects that have been reported. The identification of studies relied on a broad approach based on the terms used in the literature to describe psychosocial responses to ALAN. The selected studies were then organized and interpreted using a targeted analytical perspective informed by concepts from environmental psychology. This framework was used as a *post-hoc* interpretative grid to structure a heterogeneous corpus, rather than as an *a priori* framework guiding study selection. Accordingly, this review does not aim to provide an exhaustive account of all possible dimensions of the psychosocial processes involved in responses to ALAN, but rather to examine a specific set of psychosocial responses, processes, and determinants documented in the literature.

## 2 Method

### 2.1 Review design

Scoping reviews are an effective method for mapping the scientific literature on a specific topic (Daudt et al., 2013). This review is based on the guidelines of the Joanna Briggs Institute (Peters et al., 2024). In addition, the PRISMA-ScR (Preferred Reporting Items for Systematic Reviews and Meta-Analyses Extension for Scoping Reviews) checklist was used to write this exploratory review (Tricco et al., 2018), and the PRISMA checklist (Page et al., 2021) was used to ensure transparency and comprehensive documentation of all critical aspects. The protocol for this scoping review has been registered on the Open Science Framework (OSF No.: https://osf.io/zsbc6/).

### 2.2 Inclusion criteria

The inclusion criteria for the articles are as follows: (1) the article should examine psychosocial responses to outdoor ALAN, such as perceptions, emotions, attitudes, or behaviors, as captured by a wide range of terms used in the literature (e.g., judgement, preference, perceived safety, comfort, acceptability, intention, user experience, etc.), (2) present an empirical design, (3) be written in English. No temporal, geographical or spatial restrictions were applied when searching articles in the databases. These inclusion criteria were applied independently of the specific theoretical framing used in the studies, in order to account for the heterogeneity of terminology in the field. Only studies explicitly focusing on these aspects are included.

### 2.3 Search strategy

The search strategy was carried out in July 2025 across the following six databases: Web of Science Core Collection, APA PsycInfo, Academic Search Premier, GreenFILE, MEDLINE, and Psychology and Behavioral Sciences Collection. All databases were accessed through the EBSCO platform, with the exception of Web of Science, which was searched separately. In addition, Google Scholar was included due to its broad indexing coverage. Two filters were applied: (1) the title or abstract had to contain the selected keywords, (2) the studies included must be published in peer-reviewed journals or in gray literature. No constraint was applied to the searching period. Before conducting database searches, a preliminary phase was conducted to identify terms commonly used to refer to the psychosocial responses of outdoor ALAN. This was done in a few steps. An exploratory research phase using the snowball sampling method was conducted to identify articles that were relevant to the research question. Articles identified as pertinent were imported into the ResearchRabbit platform, as well as a short list of seminal articles well-known to the research team (Fabiano et al., 2024). A total of 581 references were identified, and nearly half were deemed to be similar. Keywords were extracted and organized according to two themes to meet the research objectives: (1) psychosocial responses; and (2) ALAN. Pilot research was carried out to assess the relevance of potential terms. These preliminary tests highlighted that generic keywords (e.g., “light”) generated a large number of non-relevant results and were therefore excluded. Approximately half of the selected keywords were verified and matched with the controlled vocabularies of the Academic Search Premier, APA PsycInfo, GreenFILE, MEDLINE, and Psychology and Behavioral Sciences Collection thesaurus and MeSH. For the remaining half, the terms were used as free-text keywords to ensure conceptual coverage across all databases. Keywords were not derived from a predefined conceptual typology, but were identified empirically from the terminology used in the literature, in order to maximize the coverage of relevant studies in a field characterized by non-standardized concepts. Keywords from each axis were combined using the Boolean operator “OR,” and the two conceptual blocks were combined using the operator “AND” to form the final search equation. The complete search strategy flowchart is presented in Box 1 and in Supplementary material S1. These databases were searched using the search equation presented in Box 1.

Box 1 **Search equation.**

(“Perception” OR “Preference” OR “Judgement” OR “Feeling of safety” OR “Emotion” OR “Mood” OR “Evaluation” OR “Reassurance” OR “Impression” OR “Intention” OR “Vulnerability” OR “Cognition” OR “Opinion” OR “Perceived adequacy” OR “Perceived restorativeness” OR “Perceived security” OR “Perceived sociability” OR “Perceived social safety” OR “Risk perception” OR “Safety perception” OR “Subjective” OR “Subjective response” OR “Well-being” OR “User experience” OR “Comfort” OR “Attitude” OR “Behavior^*^” OR “Acceptability” OR “Acceptance” OR “Public acceptability” OR “Social behavior” OR “Social judgment” OR “Social attention” OR “Social evaluation” OR “Social inference”)
  AND (“Artificial light^*^” OR “Artificial light at night” OR “Road light^*^” OR “Public light^*^” OR “Street light^*^” OR “Urban light^*^” or “Outdoor light^*^” or “Lighting control” or “Public space lighting”)

### 2.4 Study selection

The screening steps recommended by the PRISMA-ScR methodology were followed. They are presented in the flow diagram in results. The authors of potentially relevant articles were contacted. Rayyan software was used to automatically identify potential duplicates, which were then manually verified by the authors and to perform the initial selection steps based on title and abstract (Ouzzani et al., 2016). All the articles were screened manually by two reviewers (CG-C and MC). Disagreements regarding articles' inclusion were resolved by consulting a third reviewer (TP). Once the initial selection stage was complete, the remaining articles were read in full to determine their eligibility. The authors of unavailable articles were contacted. The articles excluded during the full-text review phase and the reasons for their exclusion are presented in the Supplementary material S2.

### 2.5 Quality assessment

The methodological quality of the articles included in the review was assessed using the mixed methods analysis tool (MMAT; Hong et al., 2018). Although this assessment is not required under the PRISMA-ScR guidelines (Tricco et al., 2018), it was conducted to better analyse the quality and limitations of the articles and, consequently, of the available data. The MMAT was developed to evaluate different types of studies (e.g., qualitative, randomized controlled trials, quantitative non-randomized, and mixed-method studies). This method was therefore well suited to the wide range of designs identified in the included articles. Among other things, it allows for the evaluation of potential biases and methodological rigor (Hong et al., 2018). The MMAT includes two screening questions and five criteria specific to each of the five methodological designs. The screening questions assess (1) whether the research questions are clearly formulated and (2) whether the data collected are sufficient to answer these questions. Studies that do not meet both screening questions are excluded from further evaluation. The criteria evaluate each type of study based on specific methodological requirements to each type of design (e.g., criteria 1.1. “Is the qualitative approach appropriate to answer the research question?” or criteria 2.2. “Are the groups comparable at baseline?”). Each criterion is rated on a three-point scale (Yes, No, or Can't tell). Prior to the assessment, two reviewers examined the MMAT evaluation criteria and the associated explanations in the user guide provided by the authors (Hong et al., 2018; see Supplementary material S3). Each criterion had to be rated based on the information explicitly stated in the guide. When a criterion could not be assessed due to a lack of information, it was rated “Can't tell.” The raters were instructed to evaluate each study independently and not to discuss the assigned ratings until the end of the double-coding phase. One reviewer (CG-C) systematically assessed each article using MMAT's assessment tool. To ensure reliability, 25% of the papers, selected to be representative of the included study designs, were independently double-coded by a second reviewer (MC). This method is in line with common practices in systematic reviews (e.g., Butow et al., 2020; Engel et al., 2024). Results indicated an excellent interrater reliability [ICC = 0.97, 95% CI (0.83, 1.00)] in line with Koo and Li (2016) criteria. Disagreements were resolved by consulting a third reviewer (TP). For reporting purposes, the authors applied a scoring system: “yes” received 1, “no” and “can't tell” received a 0. A maximum score of 5 for non-mixed methods studies and 15 for mixed methods studies was available. On the basis of the score obtained by each article, a quality percentage was calculated, using a cross-product where the score obtained is multiplied by 100 and then divided by the maximum value that can be obtained in the assessment (Pluye et al., 2009). These scores were included in the article to provide a concise summary of the evaluation results. The overall methodological quality of the included studies was high, with a mean MMAT score of 75.42% (range: 40% to 100%). Detailed evaluations for each study are provided in Supplementary material S4, S5.

### 2.6 Conceptual framework of psychosocial responses related to outdoor artificial lighting

Findings were analyzed using three major psychological models of environmental behavior: the Norm Activation Model (Schwartz, 1977), the Value-Belief-Norm Model (Stern, 2000), and the Comprehensive Action Determination Model (Klöckner and Blöbaum, 2010). By using this approach, it was possible to analyze the psychosocial dimensions of outdoor ALAN as an environmental phenomenon while also understanding how values, beliefs, emotions, and perceptions can influence behaviors related to the ecological and social issues of lighting. The Norm Activation Model (Schwartz, 1977) presumes that individuals are motivated by altruistic values. When aware of negative consequences for others, they assess their responsibility; this attribution, acknowledged or denied, activates behavioral norms. The Value-Belief-Norm model (Stern, 2000), is conceptually close to Schwartz's model (1977). Indeed, VBN proposes that values (altruistic, egoistic, or traditional) shape the ecological worldview, or new ecological paradigm, defined as “broad beliefs about the biosphere and the effects of human action on it” (Stern et al., 1999). This worldview influences awareness of consequences and responsibility attribution, generating moral obligation and activating pro-environmental personal norms that lead to behaviors (e.g., environmental activism, environmental citizenship, policy support, private-sphere behaviors). Finally, the Comprehensive Action Determination Model (Klöckner and Blöbaum, 2010) differs from the previous models by integrating the role of habits, intentions, attitudes and situational factors in determining behavior. Situational inferences (i.e., objective and subjective constraints) influence normative processes, such as social norms, awareness of need and awareness of consequences. They affect habits and intentions, with intentions also encompassing attitudes. These three factors (i.e., intentions, habits and situational inferences) determine behavior. Therefore, combining these three models provides a comprehensive framework that integrates perceived risks and consequences (NAM, VBN) with motivational processes, norms, values, habits, cognitions, emotions, and affective responses (VBN, NAM, CADM) and organizational constraints (CADM). This articulation allows for a structured analysis of the psychosocial dimensions of outdoor ALAN, from moral obligations and environmental beliefs to daily practices and contextual barriers influencing lighting-related behaviors.

**Figure 1:**
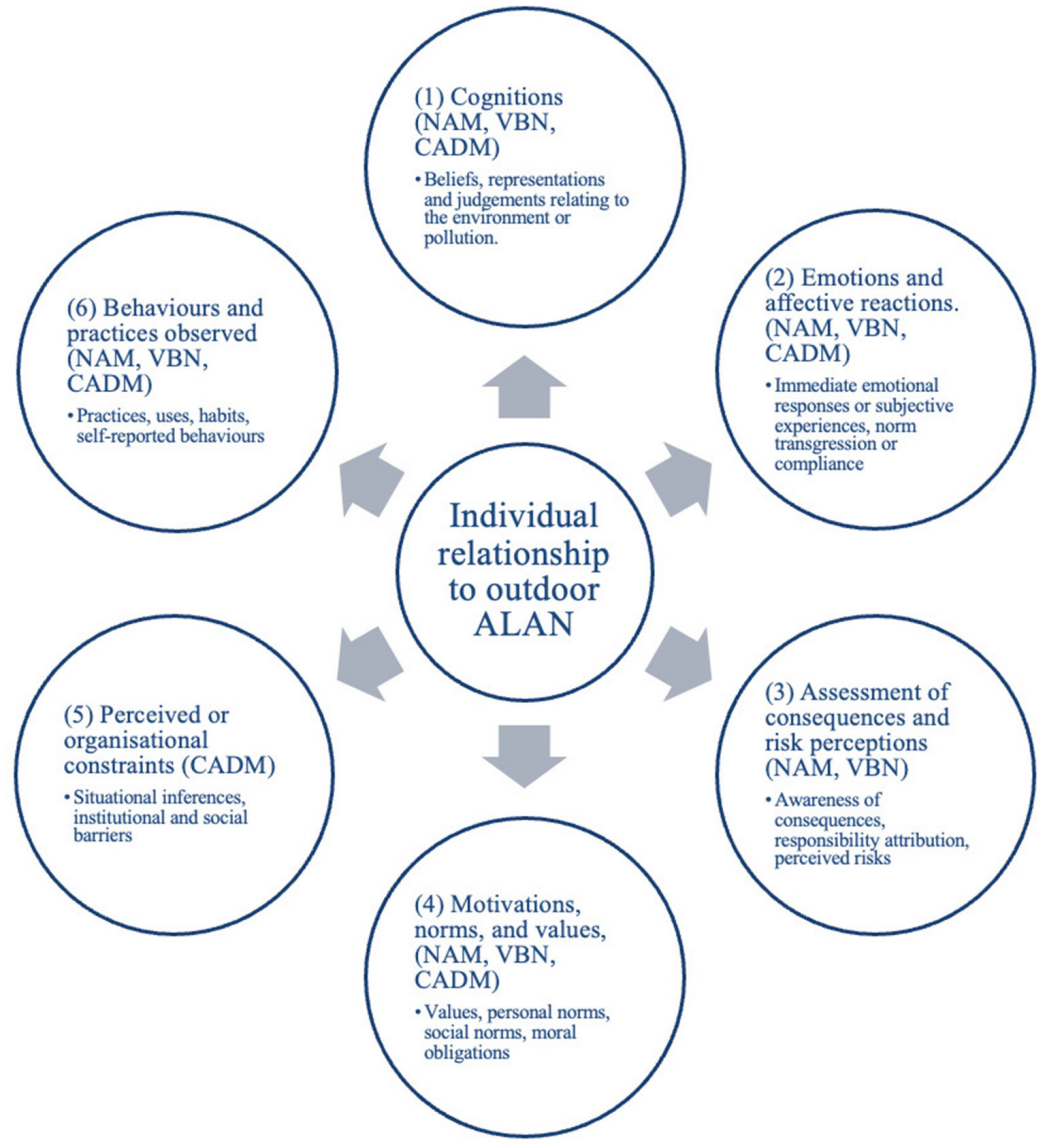
Conceptual framework of psychological determinants related to outdoor artificial lighting.

Drawing on these models, a conceptual framework comprising six psychological dimensions was developed to identify the determinants of outdoor artificial lighting: (1) cognitions; (2) emotions and affective reactions; (3) assessment of consequences and risk perception; (4) personal motivations, norms and values; (5) perceived or organizational constraints; and (6) observed behaviors and uses. The dimension (1), which refers to cognitions, includes beliefs, representations and judgements relating to the environment or pollution. In the models, this dimension refers to the ecological awareness: the altruistic values underlying NAM, the new environmental paradigm of VBN, and the cognitive processes supported by the norms and intentions of CADM. The dimension (2), emotional and affective reactions, involve immediate emotional responses or subjective experiences, corresponding to moral emotions such as shame or pride associated with norm transgression or compliance in NAM and VBN, and to affective responses arising from the adoption of pro-environmental behaviors in CADM. This dimension also encompasses immediate affective and experiential reactions (e.g., comfort, glare, impressions or preferences). Dimension (3), which deals with the assessment of consequences and perception of risk concerns the perception of risks for oneself or others due to the environment or behaviors. Unlike dimension (2), which reflects immediate emotional experience, this dimension refers to more thoughtful cognitive assessments. It reflects awareness of consequences and responsibility attribution in NAM and VBN, as well as the perception of environmental threats inherent to the new environmental paradigm of VBN. Dimension (4), related to personal motivations, norms, and values, encompasses social norms, moral judgements, and individual values. Values are central in the VBN and NAM, while norms are mentioned in all three models. The dimension (5), perceived or organizational constraints, represents psychosocial or institutional barriers to action. This refers to the situational inferences of the CADM. Finally, dimension (6) focuses on actual or self-reported behaviors by individuals. This corresponds to the habits measured in the CADM, and to the pro-environmental actions or behaviors measured in the three models (see Figure 1).

Based on this framework, it was possible to analyse the included studies. Relevant information was first extracted from each study (e.g., objectives, methods, dimension, and findings related to psychosocial responses) and systematically recorded, including the identification of relevant criteria and the corresponding page references in the original articles. The analysis then aimed to: (1) identify the presence or absence of each methodological criterion (i.e., calculate a completion percentage for each dimension, determined by the ratio between the number of studies meeting the criterion and the total number of studies for that dimension); (2) determine, for each study, whether each of the six dimensions of the conceptual framework was addressed; (3) extract all reported psychosocial findings and assign each result to the previously identified dimensions. These findings were then grouped into descriptive subcategories for readability purposes only. These subcategories do not result from a thematic or qualitative analysis but were used solely to organize similar findings under common labels (e.g., visibility, perception). For points (1) and (2), a first reviewer (CG-C) performed the categorization, which was then verified by a second reviewer (MC) based on the page references identified in the original articles; disagreements were resolved through consultation with a third reviewer (TP). For point (3), the extraction and allocation of findings to dimensions and subcategories were conducted by one reviewer (CG-C) and independently checked by a second reviewer (MC); disagreements were resolved through consultation with a third reviewer (KN).

## 3 Results

**Figure 2:**
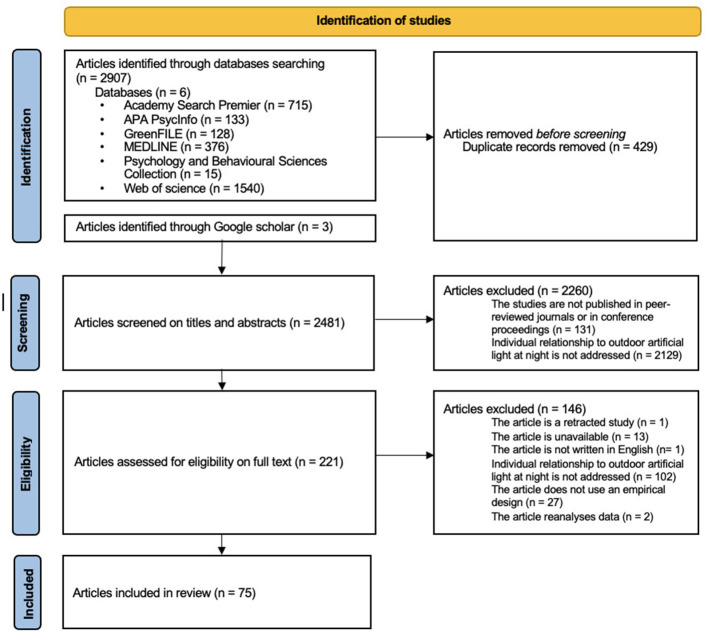
The PRISMA Flow Diagram of the systematic scoping review adapted from Moher et al. (2009).

Figure 2 illustrates the screening stages in the PRISMA flow diagram.

### 3.1 Overview and study characteristics

#### 3.1.1 Countries and publication dates

**Figure 3:**
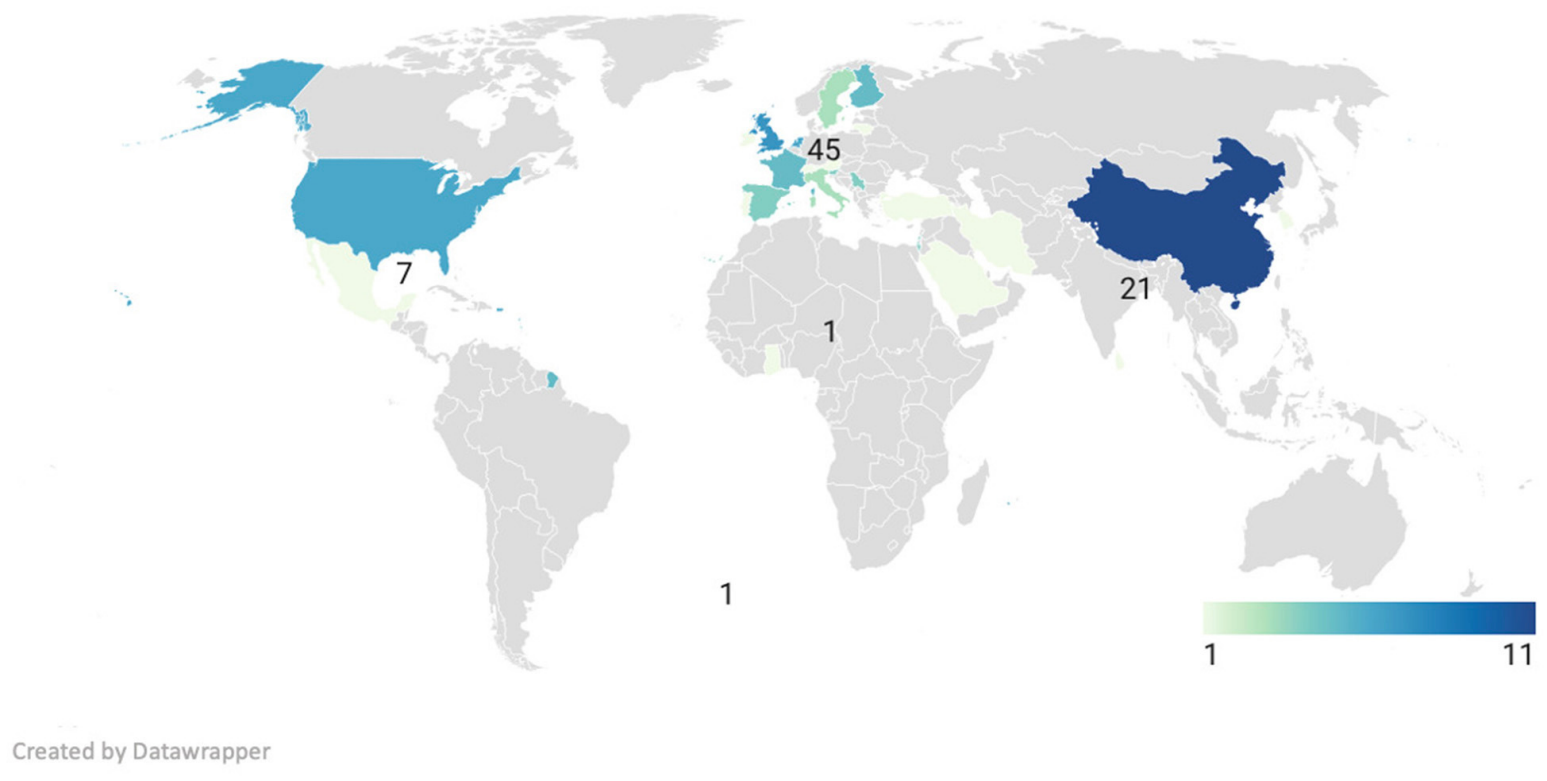
Geographical location of included studies. Note: 1 study is multinational, covering different continents.

Most of the included studies were conducted in Europe (45/75, 60%), a region largely located at mid-to-high northern latitudes where seasonal variation in daylight duration is pronounced (Forsythe et al., 1995). The largest numbers of studies were conducted in the United Kingdom (8/75, 10.7%), the Netherlands (6/75, 8.0%), Finland (5/75, 6.7%), and Sweden (3/75, 4.0%). Additional European studies were conducted in France (4/75, 5.3%), Slovenia (4/75, 5.3%), Serbia (4/75, 5.3%), Italy (3/75, 4.0%), and Spain (3/75, 4.0%). In Asia, 21 studies (28%) were identified. These studies were conducted across a broader latitudinal range, including both mid-latitude regions and areas closer to subtropical latitudes where seasonal variation in daylength is generally less pronounced (Forsythe et al., 1995). Most Asian studies were conducted in China (11/75, 14.7%) and Israel (4/75, 5.3%). Seven studies (9.3%) were conducted in North America, mainly in the United States (6/75, 8.0%). This region spans a wide latitudinal gradient, from subtropical to high-latitude regions, encompassing diverse daylight regimes across seasons (Forsythe et al., 1995). One study was conducted as a multinational collaboration between Mexico, Spain, and France (see Figure 3).

**Figure 4:**
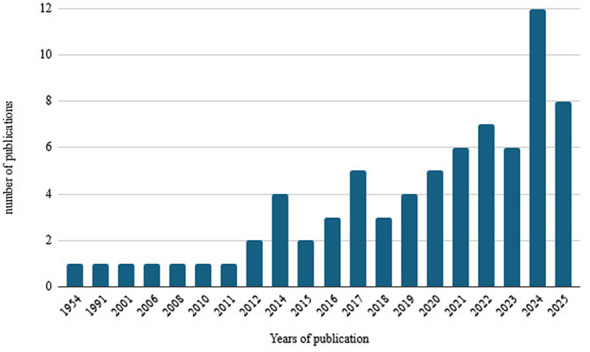
Years of publication of the included studies.

Of the 75 studies included, five were published before 2010 (6.66%), 25 between 2010 and 2019 (33.33%), and 45 (60.00%) since 2020. After a period without increase prior to 2010, the number of studies has seen a continuous increase since 2012. Figure 4 illustrates the years of publication of the articles included in this scoping review.

#### 3.1.2 Study characteristics

Among the 75 studies, 61 (81.33%) used quantitative measures, 13 mixed measures (17.33%), and one qualitative measure (1.33%). Forty-one (54.66%) studies were conducted in real-world settings, 23 (30.66%) in a controlled environment, and eight remotely (10.66%). Three studies took place in multiple contexts simultaneously (4.00%). Two of these (Bullough et al., 2008; Ferguson and Stevens, 1956) took place in real-world settings and in a controlled environment. The other one (Jayaratne et al., 2022) was conducted both in real-world settings and remotely.

#### 3.1.3 Population characteristics

Fifty-six studies indicate their target population (74.66%). Of these, two studies mention adults (2.66%), without further specification. The remaining 54 studies focus on more specific populations. Of these, 16 focused on students (21.33%), eight on drivers (10.66%), six on the local population (8.00%), five on residents (6.66%), five on pedestrians (6.66%), and three surveyed households (4.00%). Two articles surveyed women (2.66%), and students and university personnel (2.66%), respectively. The following populations are only studied in one article: national population (Orejon-Sanchez et al., 2021), visitors of a National Park (Fristrup et al., 2024), people under 30 (Wei et al., 2025b), young and old people (Rahm and Johansson, 2021), residents and students (Liu et al., 2022), citizens and shopkeepers (Meneses et al., 2016), visitors and stakeholders of an exhibition (den Ouden et al., 2012). Nineteen studies did not specify their target population (25.33%). The sample size of the 75 articles ranged from 5 (Bozorg Chenani et al., 2016) to 1,939 (Markvica et al., 2019). Six studies did not mention it.

#### 3.1.4 ALAN operationalization and measurement

Eight categories of ALAN operationalization were identified across the 75 included studies. These categories capture different ways in which ALAN is addressed across studies (e.g., measurement, manipulation, or description), rather than a single unified classification dimension. The first and most common category involves *in situ* measurements and includes twenty studies (26.67%). In these studies, existing outdoor lighting was measured objectively, without any intervention by the researchers. The second category, real-world manipulation, comprises 17 studies (22.67%). In these studies, researchers physically installed or modified lighting fixtures in the field. The third category, virtual or simulated environments, includes nine studies (12.00%). These studies presented lighting via virtual reality or simulation software. The fourth category, laboratory manipulation, comprises eight studies (10.67%). In these studies, lighting was controlled in a laboratory setting. The fifth category, unspecified existing lighting, comprises eight studies (10.67%). These studies mentioned the ALAN without any description of the lighting conditions. The sixth category, visual stimuli, comprises seven studies (9.33%). In these studies, participants evaluated photos, videos, or images. The seventh category, existing lighting described, comprises four studies (5.33%). These studies describe the lighting conditions without actually measuring them. The eighth category, “hypothetical scenario,” includes two studies (2.67%). In these studies, ALAN did not exist directly but was described through hypothetical scenarios. For more details, see Table 1.

**Table 1:** General characteristics of the included articles (*N* = 75).

References	Aim	Country	Method	ALAN operationalization	Light measurement	Study settings^a^	Artificial outdoor lighting type	Sample size, participants	Quality
Alharbi et al. (2023)	• Obtain drivers' responses on the performance of existing urban street-lighting system • Identify important criteria for performance evaluation 3) Develop a fuzzy-based evaluation method for information aggregation and decision-making.	Saudi Arabia	QT	Existing lighting unspecified	Not measured	Remotely	Urban lighting	*N* = 253, drivers	60.00
Atici et al. (2011)	Develop an intelligent road lighting system capable of sensing its environment and responding adaptively, by analyzing efficient system architecture and communication technologies while addressing user needs.	Netherlands	QT	Real-environment manipulation	Measured	Field	Campus lightscape	N/S, N/S	60.00
Beaudet et al. (2022)	Examine citizens' willingness to accept changes toward more sustainable lighting types.	France	QT	Hypothetical scenario	Not measured	Remotely	Street lighting	*N* = 1,148, local population	100.00
Boomsma and Steg (2014a)	Examined whether providing information about the environmental impact of street lighting influences the acceptability and perceived social safety of reduced street lighting levels.	Netherlands	QT	Virtual/simulated environment	Measured	Control environment	Street lighting	*N* = 88, students	80.00
Boomsma and Steg (2014b)	Examine the influence of entrapment and gender on perceived social safety and acceptability under different lighting levels.	Netherlands	QT	Virtual/simulated environment	Measured	Control environment	Street lighting	*N* = 73, students	80.00
Bordel et al. (2025)	Examine how residents of a municipality in southern France perceive a policy of reducing artificial night lighting from 1 a.m. to 5 a.m.	France	QT	Existing lighting, unspecified	Not measured	Remotely	Urban lighting	*N* = 91, residents	100.00
Bozorg Chenani et al., 2016	Effect of different lighting levels from road luminaires on drivers' visual performance on a low traffic urban road.	Finland	QT	Real-environment manipulation	Measured	Field	Road lighting	*N* = 5, drivers	60.00
Bullough et al. (2008)	Assess the relative impacts of light source photometric characteristics on subjective ratings of discomfort glare	United States	QT	Real-environment manipulation	Measured	Field and control environment	Parking and urban lighting	N/S, N/S	80.00
Burattini et al. (2025)	Investigate the effects of street luminance on pedestrians' comfort, visibility, and perception.	Italy	QT	*In situ* measurement	Measured	Field	Urban lighting	*N* = 40, N/S	80.00
(Rozman Cafuta, 2021)	Present a holistic assessment approach to evaluate the impact of artificial night lighting, by integrating users' environmental perceptions, priorities, and spatial responses, using the SEC (suitable for everyone, environmentally-accepted, cost-effective) methodology and model.	Slovenia	QT	*In situ* measurement	Measured	Control environment	Street, park, and urban lighting	*N* = 200, students	60.00
(Rozman Cafuta, 2021)	Develop and validate the durability coefficient of exterior lighting environments (Sn) to measure the luminous efficiency of urban spaces based on human perception.	Slovenia	QT	*In situ* measurement	Measured	Control environment	Street, park, and urban lighting	*N* = 200, students	60.00
Castilla et al. (2024)	Analyzing residents' affective impressions of their neighborhood lighting, based on their activities, from a citizen-centered lighting design perspective.	Spain	QT	Existing lighting, unspecified	Not measured	Field	Street, park, urban lighting	*N* = 310, residents	80.00
Chen et al. (2024)	Investigates the non-visual effects of correlated color temperature and illuminance levels of urban motor vehicle road lighting on driver alertness during various driving tasks.	China	QT	Laboratory manipulation	Measured	Control environment	Road lighting	*N* = 18, drivers	80.00
Coogan et al. (2020)	Examine public perception about light pollution.	Ireland	QT	Existing lighting, unspecified	Not measured	Remotely	Light pollution	*N* = 462, N/S	80.00
(Calvillo Cortés and Falcón Morales 2016)	Assess the emotions experienced by participants from different cultural backgrounds in outdoor public spaces.	Mexico, Spain, and France	QT	Laboratory manipulation	Measured	Control environment	Park and urban lighting	*N* = 217, students	60.00
Davidovic et al. (2019b)	Compare the subjective assessments of two street lighting colors.	Serbia	QT	Real-environment manipulation	Measured	Field	Street lighting	*N* = 53, drivers	80.00
Davidovic et al. (2019a)	Establish the driver's preferred LED light color.	Serbia	QT	Real-environment manipulation	Measured	Field	Street lighting	*N* = 139, students	100.00
(de Oliveira Grando and Ghisi 2021)	Evaluate public lighting systems installed in a concrete pathway considering the effects of mesopic vision and sense of security.	Brazil	M	*In situ* measurement	Measured	Field	Campus lightscape	*N* = 51, N/S	60.00
den Ouden et al. (2012)	Generate insights into stakeholders' perceptions of the intangible value of different lighting settings	Netherlands	M	Laboratory manipulation	Measured	Control environment	Road lighting	N/S, exhibition visitors and stakeholders	60.00
Djokic et al. (2018)	Compare drivers' subjective impressions under high-pressure sodium vs. LED street lighting.	Serbia	QT	*In situ* measurement	Measured	Field	Street lighting	N/S, students	100.00
Ferguson and Stevens (1956)	Compare observers' judgments of the apparent brightness of high-luminance mercury and sodium light sources.	United Kingdom	QT	Laboratory manipulation	Study 1: not measured	Study 1: control environment	Street lighting	N/S, N/S	40.00
					Study 2: measured	Study 2: field			
Fotios et al. (2015)	Study the influence of road lighting on emotional judgments and gaze direction.	United Kingdom	QT	Visual stimuli	Measured	Control environment	Road lighting	*N* = 30, students and university personnel	100.00
Fotios et al. (2017)	Evaluate how lamp spectrum affects the ability to judge the intentions of another person under conditions better resembling those of pedestrian behavior.	United Kingdom	QT	Visual stimuli	Measured	Control environment	Road lighting	*N* = 28, N/S	80.00
Fristrup et al. (2024)	Identify the effects of lighting color and intensity on nighttime visitor experiences in a National Park with attention to perceived benefits for both visitors and wildlife.	United States	QT	Real-environment manipulation	Measured	Field	Park lighting	*N* = 573, visitors	60.00
Girard et al. (2023)	Model the discomfort level for outdoor lighting.	France	QT	Laboratory manipulation	Measured	Control environment	Street, urban, and road lighting	*N* = 40, N/S	100.00
Hamoodh et al. (2023)	Assess if the face is the most important visual cue in the evaluation of facial identity and facial emotion evaluations under road lighting.	United Kingdom	QT	Visual stimuli	Measured	Control environment	Road lighting	*N* = 44, N/S	80.00
Hao et al. (2022)	Conduct a cross-sensory test to evaluate urban street lighting with multiple combinations of CCT values and illuminance levels according to pedestrians' visual perception and psychological preferences.	China	QT	Visual stimuli	Measured	Control environment	Urban lighting	*N* = 77, students	60.00
Hickcox et al. (2012)	Assess the effect of different spectral power distribution on perception of discomfort glare.	United States	QT	Laboratory manipulation	Measured	Control environment	Lighting technology	*N* = 10, N/S	80.00
Himschoot et al. (2024)	Examine how different artificial lighting conditions influence visitors' perceptions of safety and their potential to have pleasant experiences while recreating outdoors at night in a park-like setting.	United States	QT	Real-environment manipulation	Measured	Field	Campus lightscape	*N* = 156, students	80.00
Huang et al. (2024)	Examine lightscape perceptions and their impacts across different campuses by conducting lightscape walking experiments, subjective questionnaires, objective luminance measurements, and HDR image processing at Chongqing University's A and B campuses	China	M	*In situ* measurement	Measured	Field	Campus lightscape	*N* = 788, students	86.67
Ingi et al. (2025)	Explore the use of the public participation geographic information system methodology to examine the relationship between reassurance and outdoor road lighting, focusing on participants' everyday experiences.	Finland	QT	*In situ* measurement	Measured	Field	Campus lightscape	*N* = 111, N/S	80.00
Jayaratne et al. (2022)	Assess the current perceptions of the Sri Lankan market regarding the transition to LED lighting technologies.	Sri-Lanka	M	Existing lighting, unspecified	Not measured	Field and remotely	Lighting technology	*N* = 85, N/S	80.00
Juntunen et al. (2015)	Achieve energy savings in street lighting through advanced LED technology without compromising end-user comfort.	Finland	QT	Real-environment manipulation	Measured	Field	Road lighting	*N* = 23, pedestrians	60.00
Kim and Noh (2018)	Discuss lighting for pedestrians in urban obscured spaces and explore how perceived adequacy of illumination (PAI) can enhance pedestrians' night-time experiences.	United Kingdom	QT	*In situ* measurement	Measured	Field	Lighting under a bridge	*N* = 30, pedestrians	60.00
Knight (2010)	Examine the effect of public lighting lamp spectra on face recognition and the perception of safety and comfort in outdoor environments.	Netherlands	M	*In situ* measurement	Measured	Field	Street lighting	*N* = 356, residents	93.33
Lanini-Maggi et al. (2024)	Inform design decisions aimed at enhancing the emotional wellbeing of people outdoors at night in urban environments.	Switzerland	QT	Virtual/simulated environment	Measured	Remotely	Park lighting	*N* = 48, N/S	80.00
Li et al. (2024)	Explore the temporal and spatial characteristics of outdoor lightscapes on university campuses, identify perceptual factors influencing these lightscapes, and provide recommendations for sustainable design, planning, and management.	China	M	*In situ* measurement	Measured	Field	Campus lightscape	*N* = 51, students	93.33
Liu et al. (2020)	Understand a driver's driving speed selection behavior in low illumination.	China	M	Hypothetical scenario	Not measured	Field	Street lighting	*N* = 243, drivers	80.00
Liu et al. (2021)	Examine the relationship between drivers' risk perception and their propensity for risky driving behavior, through a comparative analysis under conditions with and without street lighting.	China	QT	Existing lighting, described	Not measured	Field	Street lighting	*N* = 266, drivers	80.00
Liu et al. (2022)	Explore the association between street lighting attributes, such as illuminance and wavelength, and pedestrians' feeling of safety (FoS) and perceived lighting quality (PLQ) in residential districts.	China	QT	Existing lighting, described	Not measured	Field	Street lighting	*N* = 103, residents and students	80.00
Madvari et al. (2023)	Investigate the interactions between mood, fatigue, mental workload, and sleepiness and their relationship with quantitative indicators of street lighting in passenger car drivers.	Iran	M	*In situ* measurement	Measured	Field	Street lighting	*N* = 270, drivers	100.00
Mao and Fotios (2025)	Evaluate the facial emotion recognition with full-face and 3/4 views.	United Kingdom	QT	Visual stimuli	Measured	Control environment	Road lighting	*N* = 30, N/S	80.00
Markvica et al. (2019)	Evaluate three different lighting scenarios under actual conditions in an urban environment and assess their influence on road user perception of public space and mobility behavior.	Austria	M	Real-environment manipulation	Measured	Field	Street lighting	*N* = 1,939, pedestrians	60.00
Masullo et al. (2022)	Investigate the influence of lighting on individuals' emotions in an outdoor environment.	Italy	QT	Virtual/simulated environment	Measured	Control environment	Park lighting	*N* = 36, students and university personnel	60.00
Meneses et al. (2016)	(1) Understand citizens' assessments of street lighting, (2) measure their knowledge about energy efficiency, (3) develop hypotheses on how street lighting energy consumption can be improved without compromising citizens' security.	Portugal	M	Existing lighting, described	Not measured	Field	Street lighting	*N* = 735, citizens and shopkeepers	80.00
Messa et al. (2025)	Evaluate women's perceived level of safety at night.	Italy	QT	Existing lighting, unspecified	Not measured	Field	Urban lighting	N/S, women	60.00
(Nikunen et al. 2014)	Explore the idea that different outdoor lighting qualities may affect the perceived restorativeness of the night-time environment.	Finland	QT	*In situ* measurement	Measured	Field	Street lighting	*N* = 55, N/S	80.00
Orejon-Sanchez et al. (2021)	Investigate several mechanisms to enhance the acceptability of autonomous solar-powered lighting (ASL) equipment in urban context.	Spain	QT	Real-environment manipulation	Measured	Remotely	Urban lighting	*N* = 453, national population	60.00
Painter and Farrington (2001)	Evaluate the impact of improved street lighting on crime.	United Kingdom	QLT	Real-environment manipulation	Measured	Field	Street lighting	*N* = 641, households	100.00
Park and Garcia (2020)	Explore the relationship between street conditions and the perception of pedestrian safety.	United States	QT	Visual stimuli	Not measured	Remotely	Urban lighting	*N* = 160, students	80.00
Patching et al. (2017)	Determine participants' preferences for four different lighting applications using the random walk procedure described, and relate the results obtained by random walking to self-report measures completed during a guided structured walk.	Sweden	QT	Real-environment manipulation	Measured	Field	Park lighting	*N* = 80, N/S	80.00
(Peña-García et al. 2024)	Analyze the human factors involved when people develop their activities under public lighting by the development of the Basic Process of Lighting (BPL).	Spain	QT	Existing lighting, unspecified	Not measured	Remotely	Urban lighting	*N* = 133, N/S	60.00
Pihlajaniemi et al. (2023)	Test a controllable LED lighting with PIR (passive infrared) presence sensors to reduce the energy consumption of road lighting while maintaining user comfort and safety.	Finland	M	Real-environment manipulation	Measured	Field	Road lighting	*N* = 1,000, households	100.00
Portnov et al. (2020)	Develop a model that links various public space lighting attributes to pedestrians' feeling of safety, while accounting for individual, locational, environmental, and temporal factors, in order to address the existing knowledge gap.	Israel	QT	Existing lighting, described	Not measured	Field	Urban lighting	*N* = 380, local population	80.00
Portnov et al. (2022)	Determine if segmented regression yields a precise estimate of the optimal illuminance for reassurance.	Israel	QT	*In situ* measurement	Measured	Field	Road lighting	*N* = 380, local population	80.00
Rahm and Johansson (2018)	Identify lighting applications suitable for all age groups.	Sweden	QT	Laboratory manipulation	Measured	Control environment	Urban lighting	*N* = 89, pedestrians	100.00
Rahm and Johansson (2021)	Assess if methods for capturing the pedestrian experience of outdoor lighting, previously evaluated in a full-scale laboratory, were applicable in a real-world setting.	Sweden	QT	Real-environment manipulation	Measured	Field	Park lighting	*N* = 81, young and old	100.00
Rakonjac et al. (2022)	Demonstrate the limitations of current outdoor lighting design standards and guidelines in enhancing the livability of open public spaces during nighttime, especially in waterfront areas.	Serbia	QT	*In situ* measurement	Measured	Field	Urban lighting	*N* = 231, local population	60.00
Rea et al. (2017)	• Validate model developed by Rea et al. (2011). • Determine how lighting design can be used to systematically reduce power densities for parking lot applications.	United States	QT	*In situ* measurement	Measured	Field	Parking lighting	*N* = 18, adults	60.00
Saad et al. (2021)	Determine the potential energy savings achievable by optimizing street lighting attributes, such as light color and uniformity, while preserving pedestrians' feeling of safety.	Israel	QT	*In situ* measurement	Measured	Field	Street lighting	*N* = 380, local population	80.00
Shahar et al. (2018)	Compare night-time driving on a country road under three conditions.	France	QT	Virtual/simulated environment	Measured	Control environment	Road lighting	*N* = 20, drivers	60.00
Sim et al. (2017)	Investigate the existing methods for evaluating discomfort glare and subjective evaluation methods related to the characteristics of light trespass.	South Korea	QT	Virtual/simulated environment	Measured	Control environment	Light pollution	*N* = 50, N/S	80.00
Son et al. (2024)	Investigate the relationship between lighting environment and fear of crime in narrow residential streets using simulated virtual reality.	South Korea	QT	Virtual/simulated environment	Measured	Control environment	Urban lighting	*N* = 100, adults	80.00
Svechkina et al. (2020b)	Develop an interactive mobile phone application to assess and report perceived street lighting attributes and feelings of safety while walking at night.	Israel	QT	*In situ* measurement	Measured	Field	Urban lighting	*N* = 106, local population	80.00
Tural and Yener (2006)	Identify the lighting needs of monuments.	Türkiye	QT	Laboratory manipulation	Measured	Control environment	Urban lighting	*N* = 113, students	40.00
Uttley et al. (2024)	Assess pedestrian reassurance with the daylight evaluation carried out at two times of the day.	United Kingdom	QT	*In situ* measurement	Measured	Field	Road lighting	*N* = 55, pedestrians	60.00
Viliunas et al. (2014)	Identify the key subjective factors influencing the assessment of luminance distribution in an intelligent LED-based outdoor lighting system designed for pedestrians	Lithuania	QT	Real-environment manipulation	Measured	Field	Campus lightscape	*N* = 21; women	60.00
Vrij and Winkel (1991)	Examine the relationship between fear and built environment characteristics.	Netherlands	QT	Real-environment manipulation	Not measured	Field	Street lighting	Study 1: *N* = 854	90.00
								Study 2: *N* =160, residents	
Wang et al. (2025)	Determine whether this new interactive lighting model can balance pedestrian safety with energy savings, compared with other lighting approaches used in low-light environments.	China	QT	Virtual/simulated environment	Measured	Control environment	Street lighting	*N* = 30, students	60.00
Wei et al. (2022)	Develop and apply a model for assessing the light comfort of residents in public spaces by combining user perception and nighttime remote sensing data.	China	QT	*In situ* measurement	Measured	Field	Urban lighting	*N* = 94, residents	80.00
Wei et al. (2025a)	Evaluate differences in perception under daytime and after dark environments with the day-dark approach to determine the optimal lighting conditions.	Slovenia	QT	*In situ* measurement	Measured	Field	Road lighting	*N* = 35, N/S	60.00
Wei et al. (2025b)	Investigate pedestrian preferences regarding outdoor public lighting, specifically examining their impact on the perception of visual comfort, safety in different urban settings.	Slovenia	QT	Virtual/simulated environment	Measured	Control environment	Urban lighting	*N* = 130, under 30 years old	60.00
Wrigley-Asante et al. (2019)	Examine perception of safety and security, factors that influence this perception and the consequences of feeling insecure in a low-income neighborhood.	Ghana	M	Existing lighting, unspecified	Not measured	Field	Urban lighting	*N* = 510, households	93.33
Zeng et al. (2025)	Examine how different landscape lighting conditions affect emotions and the perceived restorative potential, providing a mixed-method research framework to assess nighttime landscapes.	China	M	Real-environment manipulation	Measured	Field	Campus lightscape	*N* = 8, students	80.00
Zhao et al. (2023)	Consider the impact of natural light on urban green spaces for visitor's experiences.	China	QT	Visual stimuli	Not measured	Control environment	Park lighting	*N* = 78, students	40.00

QT, quantitative; QLT, qualitative; M, mixed; N/S, not specified.

^a^Study settings refers to the setting in which participants were exposed to or evaluated artificial outdoor lighting.

Regarding the objective measurement of light, 59 of the 75 studies (78.67%) reported quantitative photometric data. These measurements were obtained through physical instruments (e.g., lux meters, luminance meters, spectroradiometers), calibrated simulation software (e.g., Dialux), or controlled experimental parameters (e.g., screen luminance in cd/m^2^, VR-defined illuminance levels). The remaining 16 studies (21.33%) did not report any objective photometric measurement; in these, lighting conditions were only described qualitatively or assessed through participant self-report. These operationalizations reflect variability in how ALAN exposure is defined across studies, ranging from directly measured lighting conditions to self-reported or context-based descriptions. For more details, see Table 1.

#### 3.1.5 ALAN lighting type

Five lighting categories were identified in the 75 articles based on the terms used by the authors. The first category, the most general, refers to light pollution and is addressed by two studies (2.66%). The second category concerns studies that examine generic outdoor public lighting, without focusing on a particular context or technology. It includes 49 studies (65.33%): 19 explore psychosocial responses toward street lighting, 17 toward urban lighting, and 13 toward road lighting. Road lighting refers to lighting for roads intended for motor vehicle traffic (e.g., motorways). Street lighting refers to lighting for streets in urban contexts. When the distinction was unclear, street lamps were classified in the more general category of urban lighting. The third category addresses specific spatial contexts and includes 16 studies (21.33%). Eight of these assess campus lightscape, six park lighting, one parking lighting, and another focus on lighting under a bridge. The fourth category investigates multiple outdoor lighting contexts within the same study and is addressed by six studies (8.00%). In these, three examine street, park and urban lighting at the same time. Then, one study was carried out in each of these three contexts: (1) parking and urban lighting together, (2) park and urban lighting; (3) street, urban and road lighting. Finally, the last category refers to a specific lighting technology -LED lighting- and is supported by two studies (2.66%). For more details, see Table 1.

### 3.2 Methodological structures of the included articles and categorization within the dimensions of the conceptual framework

#### 3.2.1 Methodological structure of included articles

Of the 75 articles, 41 are experimental (54.66%), 29 are observational (38.66%), and 11 are interventional (14.66%). Seventy-one articles are cross-sectional (94.66%) and six are longitudinal (8.00%). Regarding the measures used, 69 articles use questionnaires (92.00%) and 13 use interviews (17.33%). The measures are repeated for 45 articles (60.00%). Detailed categorization for each study, including the identification of the relevant criteria and the corresponding page numbers in the original articles, is provided in the Supplementary material S6.

#### 3.2.2 Self-reported scales used to study psychosocial' responses toward outdoor ALAN

The measurement questionnaires used to characterize psychosocial responses toward ALAN include both previously used or published scales and *ad hoc* measures developed for individual studies.

Several studies have repeatedly used instruments that have been specifically published or used in the literature. The perceived quality of outdoor lighting scale by Johansson et al. (2014) appears in three articles (Patching et al., 2017; Rahm and Johansson, 2018, 2021). Four other scales are each used in two studies: the De Boer scale (De Boer, 1967; Bullough et al., 2008; Hickcox et al., 2012); the Karolinska Sleepiness Scale (KSS) (Chen et al., 2024; Madvari et al., 2023); the Positive and Negative Affect Scale by Watson and Clark (1988), Madvari et al. (2023), and Masullo et al. (2022), and the Perceived Personal Danger Scale by (Blöbaum and Hunecke 2005) and (Boomsma and Steg 2014a,b).

In addition, a range of other published scales is used once across the reviewed studies: the Recovery Capacity Perception Scale (Nikunen et al., 2014); the Geneva Emotion Wheel (Lanini-Maggi et al., 2024); the Samn and Perelli (1982) Fatigue Scale (SAMN-PERELLI) (Madvari et al., 2023); the Folstein and Luria (1973) Mood Index (Madvari et al., 2023); the Hart and Staveland (1988) Mental Load Scale, NASA-TLX (Madvari et al., 2023); the Differential Emotion Scale (Rahm and Johansson, 2018); the Illuminating Engineering Society's Glare Sensation Vote (GSV) (Sim et al., 2017); the self-assessment scales for anxiety and depression (Zeng et al., 2025); the biospheric values scale by (De Groot and Steg 2008) and Boomsma and Steg (2014b); the Situation-Response questionnaire (Calvillo Cortés and Falcón Morales, 2016); the presence sensation scale by Lessiter et al. (2001), Son et al. (2024), and (Kûller 1991) enjoyment perception scale (Patching et al., 2017).

The remaining instruments correspond to questionnaires specifically developed for individual studies. These include a questionnaire developed by Kostic and Djokic (2014) (in Davidovic et al., 2019a) which examines subjective impressions; a questionnaire designed by Djokic et al. (2018) and Davidovic et al. (2019b) which assesses drivers's color preference; a questionnaire designed by Fotios et al. (2019) and Uttley et al. (2024) which measures pedestrian reassurance; another questionnaire designed by Masullo et al. (2021, 2022) which examines individual emotions; a scale developed by Wei et al. (2025a) and Wang et al. (2025) which assesses reassurance; a scale used by Son et al. (2023) and Wang et al. (2025) which assesses fear of crime. Finally, three driving-related questionnaires are employed: Zheng (2013) Driving Skills Assessment Questionnaire (Liu et al., 2020), the Modified Driver Behavior Questionnaire (DBQ) (Liu et al., 2021), and the Driver Skills Inventory (DSI) (Liu et al., 2021).

In addition to these instruments, several studies relied on *ad hoc* measures specifically developed to assess particular aspects of individuals' perceptions or experiences of outdoor lighting. Thus, among these questionnaires, 12 examine only one dimension. Four scales have been developed to measure cognitions (Alharbi et al., 2023; Bordel et al., 2025; Coogan et al., 2020; Jayaratne et al., 2022); six addressed emotions and affective reactions (Atici et al., 2011; Bozorg Chenani et al., 2016; Girard et al., 2023; Hamoodh et al., 2023; Peña-García et al., 2024; Sim et al., 2017); one examines assessment of consequences and perception of risk (Son et al., 2024); and one study motivations, norms and values (Boomsma and Steg, 2014b). In one study, scenario-based approaches (i.e., discrete choice experiments, Louviere et al., 2000) were used rather than psychometric scales, in combination with *ad hoc* self-reported questions to assess social acceptability and experiential perceptions of outdoor lighting (e.g., Beaudet et al., 2022).

The remaining 40 scales examine several psychosocial dimensions at once, such as perception, comfort, feeling of safety, attitude, spatial use (Burattini et al., 2025; Rozman Cafuta, 2021, 2024; Castilla et al., 2024; Davidovic et al., 2019b; de Oliveira Grando and Ghisi, 2021; den Ouden et al., 2012; Djokic et al., 2018; Ferguson and Stevens, 1956; Fristrup et al., 2024; Hao et al., 2022; Himschoot et al., 2024; Huang et al., 2024; Ingi et al., 2025; Juntunen et al., 2015; Kim and Noh, 2018; Knight, 2010; Li et al., 2024; Liu et al., 2022; Markvica et al., 2019; Messa et al., 2025; Nikunen et al., 2014; Orejon-Sanchez et al., 2021; Park and Garcia, 2020; Pihlajaniemi et al., 2023; Portnov et al., 2020, 2022; Rakonjac et al., 2022; Rea et al., 2017; Saad et al., 2021; Shahar et al., 2018; Svechkina et al., 2020b; Viliunas et al., 2014; Vrij and Winkel, 1991; Wang et al., 2025; Wei et al., 2022, 2025a,b; Wrigley-Asante et al., 2019; Zhao et al., 2023). Table 2 summarizes information regarding the scales used by the included studies.

**Table 2:** Frequency and classification of measurement instruments used to characterize psychosocial responses associated with outdoor ALAN.

Instrument type	Subtype	*n*	Instruments/references
Previously used or published scales	Reused (≥2 studies)	5	Perceived outdoor lighting quality; De Boer Scale; Karolinska Sleepiness Scale; Positive and Negative Affect Scale (PANAS); Perceived Personal Danger Scale
	Single use	22	Recovery Capacity Perception Scale (PRS); Geneva Emotion Wheel; Samn–Perelli Fatigue Scale (SAMN-PERELLI); Mood Index; National Aeronautics and Space Administration Task Load Index (NASA-TLX); Differential Emotion Scale; IES Glare Sensation Vote (GSV); Anxiety and Depression Scales; Biospheric Values Scale; Situation-Response Questionnaire; Sense of Presence; Perceived Pleasantness; driving ladders [driver skills inventory (DSI), driver behavior questionnaire (DBQ), driving skills assessment questionnaire (DSAQ)]
Study-specific questionnaires	-	9	Questionnaires assessing subjective impressions, color preference, pedestrian reassurance, emotions, reassurance, fear of crime, and driving skills or behavior
*Ad hoc* measures developed for individual studies	One-dimensional	13	Cognitions (4); emotions or affective reactions (6); risk or consequence appraisal (1); motivations, norms and values (1)
	Multidimensional	40	Questionnaires combining several psychological dimensions (e.g., cognitive, affective, safety-related, fear-related, behavioral)

#### 3.2.3 Theoretical analytical framework used and mapping of psychosocial dimensions studied

No article used a theoretical analytical framework to examine psychosocial responses toward ALAN.

**Figure 5:**
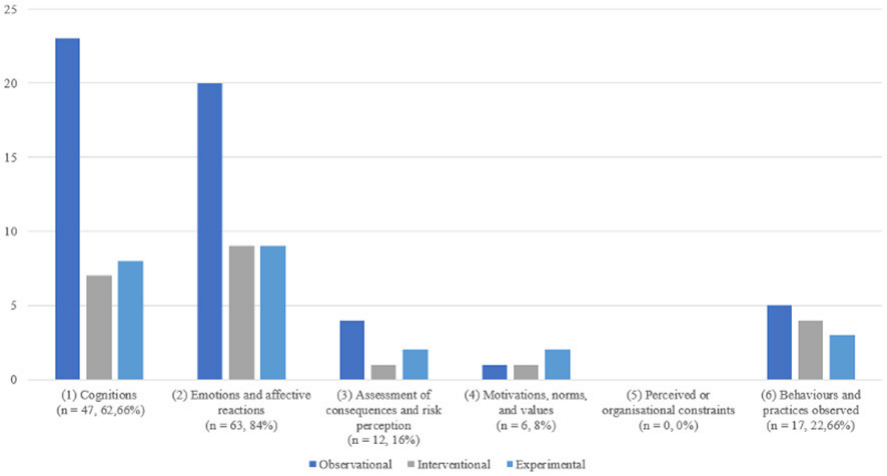
Summary of methodological structures and psychosocial dimensions examined in the included studies. Note: The percentages indicate the proportion of the 75 studies reviewed that meet each methodological criterion and psychological dimension. Studies could be classified into more than one psychological dimension; therefore, totals exceed *N* = 75. The table containing the coding pages and notes used to create this crossed table is available in the Supplementary material section.

The 75 included articles were classified according to six dimensions derived from psychological models: (1) cognitions, (2) emotions and affective reactions, (3) assessment of consequences and risk perception, (4) motivations, personal norms and values, (5) organizational constraints or perceived constraints, and (6) observed behaviors and practices. Twenty-two articles examine one dimension, while 53 examine several at once. Forty-seven articles (62.66%) focus on dimension (1) and 63 articles (84.00%) on dimension (2), which is the most studied. Twelve articles (16.00%) study dimension (3) and six (8.00%) examine dimension (4). No articles examine dimension (5). Finally, 17 articles (22.66%) explore dimension (6). Detailed categorization for each study, including the identification of the relevant criteria and the corresponding page numbers in the original articles, is provided in the Supplementary material S6. Figure 5 summarizes all the information presented concerning the methodological structure of the included articles and the dimensions studied.

### 3.3 Findings of included articles

Table 3 presents the results reported in the studies regarding psychosocial responses to ALAN. When multiple studies reported similar results, these were grouped together and synthesized into a single finding, while retaining the associated references. Finally, the results were organized according to the six dimensions of the conceptual framework.

**Table 3:** Findings of the included articles (*N* = 75).

Dimensions	Subcategories	Findings: (+) opportunities, (–) challenges, (=) neutral or descriptive observation, (±) ambiguous result, and (ø) lack of data.
(1) Cognitions		
	Perception	• (=) Overall perception is affected by artificial night-time lighting (Rozman Cafuta, 2021), the color temperature of urban street lighting, and also by the interaction between correlated color temperature, illuminance, and ambient temperature (Hao et al., 2022). • (=) Overall lightscape perception is affected by temporal and spatial factors (Li et al., 2024). • (=) Lightscape perception is influenced by perceived intensity and preference for lightscape types (Li et al., 2024). • (=) Circadian lightscape perception is influenced by the quantity and composition of outdoor spaces, vegetation, small-scale landscape design, and public services (Huang et al., 2024). • (=) The assessment of intelligent lighting installation is influenced by subjective wellbeing and by physical attributes of the environment (Viliunas et al., 2014). • (=) Evaluation of perceived pleasantness and liveliness is linked to horizontal illuminance (Kim and Noh, 2018). • (+) Spaces with high perceived adequacy of illumination are evaluated more positively in terms of pleasantness, liveliness, and spatial suitability (Kim and Noh, 2018). • (±) In conditions of low or high light levels, the perceived quality of lighting depends mainly on illuminance and uniformity (Liu et al., 2022). • (±) Under high illuminance, the perceived quality of lighting depends on glare and color temperature (Liu et al., 2022). • (=) Artificial lighting in urban spaces is perceived as high quality when daytime and night-time perceptions and uses of the space are very similar (Rozman Cafuta, 2021). • (=) Aesthetic preferences, perceived interest and attractiveness of urban green spaces under moonlight and artificial light are similar (Zhao et al., 2023). • (=) Perceived restorativeness is associated with the perceived pleasantness of a lighting environment (Nikunen et al., 2014), and depends more on well-designed landscapes than street lighting alone (Zeng et al., 2025). • (+) To be perceived as energy-efficient and environmentally friendly, street lighting must evoke a sense of innovation (Castilla et al., 2024). • (+) Red lighting is perceived as more protective of the environment (Fristrup et al., 2024). • (=) The sustainability of street lighting is linked to psychological and aesthetic-functional factors (Rozman Cafuta, 2024). • (±) The use of photovoltaic in urban lighting is perceived as sustainable, but power outages caused by insufficient energy generation are perceived as problematic (47%) (Orejon-Sanchez et al., 2021). • (=) Perceptions of light pollution (its presence and impacts) differ between rural and urban areas (Coogan et al., 2020). • (=) Older respondents are more likely to report the effects associated with light (e.g., wildlife behavior) (Coogan et al., 2020).
	Belief	• (+) Citizens have positive beliefs about public street lighting (Meneses et al., 2016).
	Knowledge	• (+) Citizens are aware of environmental issues (Meneses et al., 2016). • (+) Scientific knowledge influences attitudes toward outdoor ALAN impacts (Bordel et al., 2025).
	Opinion	• (=) According to road users' opinions, the performance of urban street-lighting systems is mainly affected by the positioning of electronic display panels, glare from oncoming vehicles and poor lighting quality in adverse weather conditions (Alharbi et al., 2023).
	Choice	• (=) Customer choices in artificial lighting are affected by cost, size, limited LED knowledge, low involvement of governing bodies, and individual perceptions of the technology (Jayaratne et al., 2022).
(2) Emotions and affective reactions		
	Alertness	• (+) Lighting interventions can increase alertness and reduce driver fatigue and mental workload (Madvari et al., 2023). • (±) Driver alertness is influenced by interactions between correlated color temperature and illuminance, and between correlated color temperature and task type (Chen et al., 2024). • (+) A light intensity of 30 lx and a correlated color temperature between 4,000 and 5,000 K improve driver alertness (Chen et al., 2024).
	Glare	• (±) Discomfort glare is affected by the light source, ambient, and surrounding illuminances levels (Bullough et al., 2008). • (–) Glare perception is stronger in spaces with low light intensity or a high proportion of blue light (Wei et al., 2022). • (=) Discomfort glare due to light trespass through the window varied little between sitting and lying positions, whether oriented perpendicular or parallel to the window (Sim et al., 2017). • (±) Under identical lighting conditions, discomfort from glare varies from one individual to another (Girard et al., 2021). • (+) Parking lot lighting with lower power density (Rea et al., 2017), sodium lighting (vs. mercury street lighting; Ferguson and Stevens, 1956), 3,000 K LEDS (vs. 4,000 K LEDs; Davidovic et al., 2019a,b), white or yellow luminous backgrounds (vs. blue luminous backgrounds) are less glaring (Hickcox et al., 2012). • (–) Significant glare is associated with a perception of a less restorative environment (Nikunen et al., 2014). • (=) Perceived adequacy of illumination and illuminance level is not linked with discomfort glare (Kim and Noh, 2018).
	Comfort	• (=) Lighting design influences users' comfort (Rakonjac et al., 2022). • (=) Pedestrian walking comfort is influenced by the overall luminance of pavements, lateral walls, and other pedestrians (Burattini et al., 2025). • (+) Areas with higher light intensity (Wei et al., 2022) and LED street lighting (Markvica et al., 2019), and white light (vs. yellow light) are more comfortable (Knight, 2010). • (+) Cold white lighting LED (3,912 K, high S/P) located in a green area was perceived as the most comfortable, pleasant during random walking (Patching et al., 2017). • (–) LED lighting application (3,810 K; 1.48 S/P) was judged as the least comfortable (Rahm and Johansson, 2018). • (=) When light intensity is high, individuals are more attentive to the comfort provided by the lighting (Wei et al., 2022).
	Wellbeing	• (–) Women felt less relaxed than men after the night-time walks under white street lights, with or without blue luminescent pavement on the paths (Lanini-Maggi et al., 2024). • (=) Relaxation experienced in urban green spaces under moonlight and artificial light was similar (Zhao et al., 2023).
	Visibility	• (=) Luminance influences the visibility of urban and pedestrian areas (Burattini et al., 2025). • (±) Individuals perceive a low light level with a high color rendering in the same way as a high light level with a low color rendering (den Ouden et al., 2012). • (–) Road lighting alone cannot guarantee target visibility (Bozorg Chenani et al., 2016). • (–) In the event of glare, the level of road lighting has no effect on drivers' visibility (Bozorg Chenani et al., 2016). • (=) Visibility supported by road lighting and vehicle headlights is influenced by glare from oncoming traffic Bozorg Chenani et al., 2016). • (=) Drivers' target visibility is influenced by its position relative to road lighting (Bozorg Chenani et al., 2016). • (=) The subjective evaluation of a target's visibility by drivers corresponds to objective visibility assessments, particularly regarding contrast (Bozorg Chenani et al., 2016). • (+) Pedestrians' brightness evaluations were higher for the LED lighting application (3,810 K; 1.48 S/P) than for a ceramic metal halide luminaire and another LED (Rahm and Johansson, 2018). • (+) LED lighting application (3810K; 1.48 S/P) was associated with better performance on visual tasks (e.g. facial recognition, sign reading, obstacle detection) (Rahm and Johansson, 2018). • (+) S/P lamps (de Oliveira Grando and Ghisi, 2021) and presence-sensitive lighting provide better visibility (Pihlajaniemi et al., 2023). • (+) White light is perceived as brighter (Knight, 2010) and allows better visibility and sharpness of objects and people (Meneses et al., 2016). • (±) 3,000 K LEDs are preferred to 4,000 K LEDs for detection of small dark obstacles and visibility (Davidovic et al., 2019a,b). • (±) 4,000 K LEDs are preferred to 3,000 K LEDs for distinguishing pedestrians and detecting small light-colored obstacles (Davidovic et al., 2019a,b). • (+) Pedestrians performed better at identifying facial expressions and reading road signs at longer distances under higher illuminance and more uniform light distribution (Rahm and Johansson, 2021). • (+) The identification of facial expressions, body posture, and traffic signs is facilitated by high luminance and uniformity and larger targets (Rahm and Johansson, 2021).
	Impression	(±) The sensations generated by street lighting must be consistent with the nature of urban social activities in these spaces (Castilla et al., 2024).
	Emotion	(+) Car drivers' mood improves as illuminance rises (Madvari et al., 2023). (±) An individual's mood predicts how park lighting affects them (Masullo et al., 2022). (=) The level of disability glare is associated with mood (Madvari et al., 2023). • (=) Urban lighting influences emotions (Calvillo Cortés and Falcón Morales, 2016). • (=) No difference was found between the emotional states of people walking at night under standard white street lighting and those walking on blue self-luminous pavement (Lanini-Maggi et al., 2024). • (±) The experience of certain emotions under urban lighting (pleasant surprise; inspiration; unpleasant surprise; contempt; disappointment) depends on the cultural context, unlike others (uncertainty, fear, affection, fascination, and entertainment) (Calvillo Cortés and Falcón Morales, 2016). • (=) In green spaces, the association between lightscape sources (meteorological, architectural, traffic) and the emotional evaluation is stronger (Li et al., 2024). • (+) Low or medium illuminance combined with an intermediate color temperature has a positive effect on individuals' emotions (Masullo et al., 2022). • (+) Images with clear visual information of the urban space evoked more positive emotions, and vice versa (Bozorg Chenani et al., 2016). • (–) High intensity cool light makes people feel more nervous (Masullo et al., 2022). • (–) Warm light induces more fatigue and less motivation to explore the park (Masullo et al., 2022). • (+) An intermediate correlated color temperature at low or medium illuminance positively affects individuals' feelings (Masullo et al., 2022).
	Preference	• (+) Individuals feel safer, more in control and comfortable under illuminated conditions (Shahar et al., 2018). • (+) Individuals preferred high light levels (den Ouden et al., 2012). • (±) Individuals prefer high-intensity lighting in the evening and low-intensity lighting during the night and early morning (Meneses et al., 2016). • (+) Parking lot lighting with lower power density was rated more positively in terms of lighting perceptual aspects, including both glare and perceived safety (Rea et al., 2017). • (=) Participants' preferences for light color are strongly related to comfort and overall evaluation (Hao et al., 2022). • (+) Individuals prefer warm white light LED (over neutral white; Davidovic et al., 2019a). • (+) 3,000 K LEDs are preferred to 4,000 K LEDs for their light intensity, color (Davidovic et al., 2019a,b). • (±) High ambient temperatures are associated with a preference for street lighting with medium and high correlated color temperature, and a perception of warmer light color (Hao et al., 2022). • (+) Downlighting is preferred to uplighting for monuments (Tural and Yener, 2006). • (+) High luminaires with high illuminance uniformity (Wei et al., 2025b) and red color are perceived as safer, more comfortable (Fristrup et al., 2024). • (+) Individuals preferred the experimentally designed energy-efficient street lamp (vs. commercial luminaires; Juntunen et al., 2015). • (+) Individuals preferred presence-sensitive lighting as a permanent solution (Pihlajaniemi et al., 2023). • (+) Pedestrians' strength quality evaluations are higher for the LED lighting application (3,810 K; 1.48 S/P) than for a ceramic metal halide luminaire and another LED (Rahm and Johansson, 2018). • (±) In normal regime, LED lights are preferred by drivers, whereas in mesopic regime high-pressure sodium lamps are preferred (Djokic et al., 2018).
	Interpersonal evaluation	• (+) The face is a better visual clue than the hands in determining the intentions of other pedestrians at night under urban lighting (Hamoodh et al., 2023). • (+) Judgements of others' intentions (based on facial recognition, body posture and gaze direction) are improved under higher luminance levels and with larger target sizes (Fotios et al., 2015). • (+) Facial recognition is supported by horizontal illuminance, spaces with high perceived adequacy illumination (Kim and Noh, 2018). • (+) Pedestrians' ability to recognize other people in poorly lit conditions is enhanced by dynamic public lighting (Wang et al., 2025). • (=) Emotion recognition of other intentions is easier in full-face view than in 3/4 view under road lighting (Mao and Fotios, 2025). • (–) Difficulties in emotion identification in 3/4 view are not offset by higher luminance (Mao and Fotios, 2025). • (=) Emotion recognition from facial expression is not influenced by lamp spectral power distribution or image color (Fotios et al., 2017).
	Sensory modalities	• (=) At night, sensory modalities have little influence on individuals' perception of illuminated environments, except for vision, whereas during the daytime, perception is mostly influenced by sound (Huang et al., 2024). • (+) Pedestrians' arousal evaluations were higher for the LED lighting application (3,810 K; 1.48 S/P) than for a ceramic metal halide luminaire and another LED (Rahm and Johansson, 2018).
	Experience	• (–) Individuals' night-time experiences (sensations, spatial perception, perceived suitability of lighting) in uncontrolled environments do not appear to be improved by brighter lighting (Kim and Noh, 2018). • (+) Individuals preferred their lighting experience (beauty, safety, relaxation, willingness to visit, interest) under artificial light compared with both half- and full-moonlight conditions (Zhao et al., 2023). • (+) Individuals preferred their lighting experience (safety, relaxation, willingness to visit, interest) under artificial light compared with half-moonlight conditions (Zhao et al., 2023).
	Satisfaction	• (+) Satisfaction with the perceived lighting is enhanced by optimal night-time illuminance (Huang et al., 2024). • (+) Lighting quality is perceived as satisfactory at light levels of 25–35 lx and color temperature of 4,000–5,500 K (Liu et al., 2022).
(3) Assessment of consequences and risk perception		
	Reassurance	• (+) The presence of lighting reinforces reassurance (Ingi et al., 2025). • (=) Pedestrian reassurance under road lighting is not affected by the time of the day (Uttley et al., 2024). • (+) Minimum illuminance is the lighting parameter that best predicts pedestrian reassurance (Wei et al., 2025a). • (±) The relationship between pedestrians' reassurance and illuminance depends on illuminance levels (Portnov et al., 2022). • (+) Individuals' reassurance can be enhanced by a dynamic tracking lighting mode (Wang et al., 2025).
	Safety	• (+) Individuals feel safer under artificial light conditions than under moonlight (Zhao et al., 2023). • (+) Adequate street lighting plays an important role in improving pedestrians' sense of safety (Park and Garcia, 2020). • (=) Street lighting availability influences individuals' feeling of safety (Wrigley-Asante et al., 2019). • (=) Lighting design influences individuals' sense of safety (Rakonjac et al., 2022). • (±) Feeling of safety is affected by locational, socio-demographic, temporal, and environmental factors (Portnov et al., 2020). • (±) The level of lighting needed for citizens to feel safe differs from city to city (Svechkina et al., 2020a,b). • (=) Public lighting shapes the relationship between the built environment and perceived safety (Messa et al., 2025). • (+) Individuals feel safer under warm and uniform lighting, with illumination levels between 5 and 17 lx (Liu et al., 2022). • (+) LED lamps (de Oliveira Grando and Ghisi, 2021), parking lot lighting with lower power density (Rea et al., 2017), white light (over yellow light) are perceived as safer (Knight, 2010). • (±) A progressively increasing brightness pattern is perceived safer under static street lighting, whereas under dynamic lighting conditions, individuals prefer a triangular distribution (the nearest lamppost providing the most intense light) (Atici et al., 2011). • (=) Feelings of safety are more strongly associated with light level or intensity than with light color (Himschoot et al., 2024). • (–) Quiet, deserted and low lighted places are perceived as unsafe (Vrij and Winkel, 1991). • (=) Individuals are more attentive to safety provided by lighting when light intensity is high (Wei et al., 2022). • (+) People feel safe at a lighting threshold of 5 to 10 lx, with no improvement beyond that (Svechkina et al., 2020a,b). • (+) High illuminance levels and warm lighting (compared to cooler or lower-intensity lighting) increase feelings of safety (Portnov et al., 2020; Himschoot et al., 2024). • (–) Individuals feel less safe when the light is blue (Saad et al., 2021). • (+) Warmer and more uniform street lighting can reduce energy consumption while maintaining an adequate level of safety (Saad et al., 2021). • (+) The perceived safety produced by lighting appears to be an important factor in psychological and attentional restoration (Nikunen et al., 2014). • (+) Individuals who feel a greater sense of security, regardless of the type of lighting, are more likely to report a pleasant experience, and conversely (Himschoot et al., 2024). • (=) Perceived lighting parameters (e.g., perceived overall quality, brightness) are associated with reassurance: lighting parameters are evaluated more positively in areas perceived as safe (Ingi et al., 2025). • (–) Women feel more unsafe when walking alone at night (Wrigley-Asante et al., 2019). • (–) Environments that are poorly lit and pose a greater risk of becoming trapped are perceived as less safe, especially by women (Boomsma and Steg, 2014b).
	Fear of crime	• (±) Fear of crime is influenced by natural light, streetlamps, and interior building lighting (Son et al., 2024). • (+) Improving street lighting reduces self-reported fear of crime (Painter and Farrington, 2001; Vrij and Winkel, 1991), youth delinquency (Painter and Farrington, 2001), risk of victimization, and increases the expectation of help from passers-by Vrij and Winkel (1991).
(4) Motivations, norms, and values		
	Values	• (+) Citizens consider public street lighting very important (Meneses et al., 2016). • (+) Citizens may support changes in public lighting if they are intended to save energy and environmental purposes (Beaudet et al., 2022).
	Attitude	• (±) Attitudes toward lighting measures and impacts of outdoor ALAN influence policy support (Bordel et al., 2025). • (+) Citizens are open to changes in favor of more sustainable public lighting (Beaudet et al., 2022). • (–) Any extension of public lighting beyond sunrise and sunset hours is perceived as unnecessary (Meneses et al., 2016).
	Acceptance	• (+) Individuals who recognize the negative impacts of ALAN are more likely to accept measures to reduce it (Bordel et al., 2025). • (–) Individuals who mainly perceive the positive impacts of ALAN lighting are more reluctant to accept reduction measures (Bordel et al., 2025). • (–) Acceptance of LED solutions among customers was lower than that of other lighting options (Jayaratne et al., 2022). • (+) LED lamps, and lamps with a high S/P ratio are more accepted (de Oliveira Grando and Ghisi, 2021). • (+) Neutral-white LEDs have been more widely accepted than yellow HPS lamps (Meneses et al., 2016).
	Acceptability	• (+) The vast majority of citizens support measures to reduce public lighting (Beaudet et al., 2022; Meneses et al., 2016). • (+) Turning off public lighting between 1 a.m. and 5 a.m. is generally accepted, especially in periurban areas (Beaudet et al., 2022). • (–) Citizens refuse radical changes to street lighting timetables (Meneses et al., 2016). • (–)(–) Citizens reject lighting solutions that interfere with their daily routines (Meneses et al., 2016). • (–)(–) Higher population density is associated with lower acceptability of lighting policies (Beaudet et al., 2022). • (+) Value-consistent information about the environmental benefits of reduced street lighting can increase the acceptability of the policy measure (Boomsma and Steg, 2014a). • (=) The feeling of security seems to play a role in the acceptability of changes to public lighting (Beaudet et al., 2022), and also influences the acceptability of lighting levels (Boomsma and Steg, 2014b). • (=) Regarding lighting change, the human factor (fear of crime and assaults) is prioritized over the physical aspects of lighting (Peña-García et al., 2024). • (–) Citizens who are reluctant to change in public lighting primarily cite security concerns (Beaudet et al., 2022). • (–) Citizens reject lighting solutions that compromise their safety (Meneses et al., 2016). • (–) Poorly lit environments with a higher risk of becoming trapped are perceived equally by men and women as less acceptable (Boomsma and Steg, 2014b).
(5) Perceived or organizational constraints		
		• (ø) No article examines the perceived or organizational constraints related to artificial outdoor lighting.
(6) Behaviors and practices observed		
	Use of space	• (=) Lighting design affects overall open public space usage during nighttime (duration, frequency, activities, individuals' spatial distribution) (Rakonjac et al., 2022). • (+) LED lamps provided better spatial orientation (de Oliveira Grando and Ghisi, 2021).
	Visit frequency	• (=) Visit frequency affects mental states and environmental perception of lightscapes (Huang et al., 2024). • (±) A higher visit frequency improves the perceptions of the environmental spatial atmosphere, and slightly decreases emotional, light, and social propensity evaluations of the lightscape (Huang et al., 2024). • (+) Individuals are more willing to visit urban green spaces under artificial light than under moonlight (Zhao et al., 2023).
	Behavior	• (=) Speed behavior is associated with gender, driving experience, the number of nights spent driving each week and the average annual mileage (Liu et al., 2020). • (=) Significant differences were observed in drivers' errors, lapses, risk perception between conditions with and without street lighting (Liu et al., 2021). • (=) Drivers' speed behavior is influenced by their ability to drive in low-light conditions (Liu et al., 2020). • (+) Driving speed in a street-lit environment at night has a positive effect on speed selection in low-light conditions (Liu et al., 2020). • (=) A driver's speed in an environment dimly lit by street lighting is positively associated with their driving speed in similar conditions but without streetlights (Liu et al., 2020). • (±) When light is dim and street lighting is absent, technical driving skills tend to increase speed behaviors, while the ability to perceive risk tends to reduce it (Liu et al., 2020). • (=) On a road with a speed limit of 120 km/h, driving speed is largely influenced by driving ability in low-light conditions (Liu et al., 2020). • (–) The ability to perceive risk reduces driving speed on roads with speed limits of 80 and 120 km/h (Liu et al., 2020). • (+) The adoption of risky driving behavior in low-light conditions is lower among elderly and experienced drivers (Liu et al., 2021). • (=) Presence-sensitive lighting and conventional lighting offer the same driving experience (pleasantness, uniformity, glare) (Pihlajaniemi et al., 2023). • (±) 89.40% of individuals walk less than 30 min per week after dark (Park and Garcia, 2020). • (=) Neither the type of luminaire (i.e., fluorescent tube luminaires, commercial LEDs, or optimized LEDs) nor the color rendering of warm white LEDs has any effect on pedestrian behavior (Markvica et al., 2019; Rahm and Johansson, 2021).

ALAN, artificial light at night; LED, light-emitting diode; lx, lux (unit of illuminance); K, Kelvin (unit of correlated color temperature); S/P, scotopic/photopic.

As regards dimension 1 (cognitions), perceptions constitute the main focus of research. The results show, in particular, that overall perception is affected by artificial night-time lighting (Rozman Cafuta, 2021), the color temperature of urban street lighting, as well as by the interaction between correlated color temperature, illuminance and ambient temperature (Hao et al., 2022). Overall lightscape perception is also affected by temporal and spatial factors, and by perceived intensity and preference for lightscape types (Li et al., 2024). The perception of circadian lightscape, for its part, is influenced by the quantity and composition of outdoor spaces, vegetation, small-scale landscape design, and public services (Huang et al., 2024). Some articles focus on perceptions of a particular type of lighting. The assessment of intelligent lighting installation is influenced by subjective wellbeing and by physical attributes of the environment (Viliunas et al., 2014). Other articles examine the affective perceptions and perceived quality of lighting according to its technical properties. Regarding affective concerns, evaluation of perceived pleasantness and liveliness is linked to horizontal illuminance (Kim and Noh, 2018). Spaces with high perceived adequacy of illumination are evaluated more positively in terms of pleasantness, liveliness, and spatial suitability (Kim and Noh, 2018). Regarding quality lighting evaluations, in conditions of low or high light levels, the perceived quality of lighting depends mainly on illuminance and uniformity (Liu et al., 2022). Under high illuminance, the perceived quality of lighting depends on glare and color temperature (Liu et al., 2022). Some articles focus on environmental perceptions. For example, red light is perceived as more environmentally friendly (Fristrup et al., 2024). The findings relating to this first dimension also show that citizens generally hold positive beliefs regarding street lighting and are aware of environmental issues (Meneses et al., 2016). Individuals' scientific knowledge also appears to influence attitudes toward the impacts of outdoor ALAN (Bordel et al., 2025). Finally, the perceived performance of urban public lighting is influenced by contextual factors (e.g., positioning of electronic display panels, glare from oncoming vehicles, weather conditions; Alharbi et al., 2023).

Main findings for dimension 2 (emotions and affective reactions) reveal that individuals feel safer, more in control and comfortable in the presence of lighting at night (Shahar et al., 2018). As a result, several studies have sought to characterize the levels of lighting that maximize user satisfaction. Optimal conditions have been reported between 25 and 35 lx illuminance and a color temperature of 4,000 K to 5,500 K (Liu et al., 2022). Similarly, a light intensity of 30 lx and a color temperature of 4,000 to 5,000 K improve driver alertness (Chen et al., 2024) and lighting interventions can increase alertness and reduce driver fatigue and mental workload (Madvari et al., 2023). Although this satisfaction level can be identified, no clear trend emerges regarding other characteristics of a lightscape. For instance, areas with intense lighting (Wei et al., 2022) and LED street lighting (Markvica et al., 2019) are perceived as more comfortable. Similarly, high luminaires with high illuminance uniformity (Wei et al., 2025b) and red-colored lighting (Fristrup et al., 2024) are perceived as more comfortable and safer. However, white light is perceived as brighter and more comfortable (Knight, 2010), and offers better visibility and sharpness (Meneses et al., 2016). Furthermore, LED lighting application (3,810 K; 1.48 S/P) is associated with better performance in visual tasks (e.g., face recognition, sign reading, obstacle detection; Rahm and Johansson, 2018). These disparate preferences make it challenging to design suitable lighting. Given that uncomfortable glare depends on both environmental conditions (e.g., light source, ambient and surrounding illuminance levels; Bullough et al., 2008) and personal sensitivity (Girard et al., 2021), individual differences in the perception of glare and its emotional responses contribute to the complexity of its assessment. Glare perception is stronger in spaces with low light intensity or a high proportion of blue light (Wei et al., 2022). Lighting design also plays a role in the subjective evaluation and in the appropriation of spaces. Indeed, the sensations generated by street lighting must be consistent with the social activities performed in these spaces (Castilla et al., 2024). Spaces with adequate lighting are perceived as more pleasant, livelier, more suited to spaces and facilitate facial recognition (Kim and Noh, 2018). Some studies also show that emotional responses to lighting are influenced by contextual factors, the characteristics of the lighting itself, as well as by users' initial mood, which can affect how lighting environments are perceived (Masullo et al., 2022; Calvillo Cortés and Falcón Morales, 2016). Wellbeing responses further differ across user groups: women report feeling less relaxed than men after night-time walks under white street lighting (Lanini-Maggi et al., 2024), pointing to gendered affective reactions that have received little attention so far. Designing lighting that considers both individual preferences and the context in which it is used is now even more complicated due to current cost-cutting constraints. Several strategies are being studied to address these challenges, starting with modifying lighting parameters. Adjustments in color rendering allow a reduction in lighting levels without compromising users' experience. Indeed, low lighting levels with high color rendering are perceived similarly to higher lighting levels with lower color rendering (den Ouden et al., 2012). Further solutions aim to replace the existing streetlights with more economical models. For example, presence-detecting lighting improves visibility and has no negative effect on driving (Pihlajaniemi et al., 2023). Energy-efficient streetlights are also being considered, especially since they are preferred by individuals compared to commercial luminaires (Juntunen et al., 2015). Overall, these results highlight the importance of emotional responses in the acceptance of outdoor lighting. Perceptions of safety, comfort, and visual ease seem to guide individuals' assessments of nighttime environments. Changes in outdoor lighting, particularly those related to reducing outdoor lighting, are therefore more likely to be accepted when they preserve positive affective experiences despite lower lighting levels.

The third dimension, assessment of consequences and perception of risk, showed that the presence of lighting enhances the feeling of safety (Ingi et al., 2025). The feeling of safety is influenced by lighting parameters. The level of intensity is the most associated with the feeling of safety, rather than its color (Himschoot et al., 2024). Nevertheless, high lighting levels do not generate a strong sense of security (Svechkina et al., 2020b). Individuals feel safe at a lighting threshold of 5 to 10 lx, with no improvement beyond that (Svechkina et al., 2020b). Reassurance also depends on lighting parameters. Minimum illuminance appears to be the lighting parameter that best predicts pedestrian reassurance (Wei et al., 2025a). The feeling of safety is also affected by other determinants, such as locational, socio-demographic, temporal, and environmental factors (Portnov et al., 2020). For example, the level of lighting needed for citizens to feel safe differs from city to city (Svechkina et al., 2020a,b). Moreover, familiarity with and long stay within the neighborhood reduces perceived insecurity (Wrigley-Asante et al., 2019). Gender appears to be an additional determining factor. Women report feeling less safe when walking alone at night (Wrigley-Asante et al., 2019). Furthermore, poorly lit environments where the risk of becoming trapped is higher are perceived as less safe, particularly by women (Boomsma and Steg, 2014b). It is also worth noting that people who experience a greater sense of safety, regardless of the type of lighting, are more likely to report a pleasant experience, and conversely (Himschoot et al., 2024). This finding suggests a bidirectional relationship between perceived safety and affective quality, linking this dimension to the previous one. Considering these factors can lead to several benefits to improve citizens' safety and their global quality of life. Improving street lighting (e.g., through increased illuminance levels, installation of additional lamp posts, and replacement of luminaires with higher-output systems) can reduce self-reported youth delinquency (Painter and Farrington, 2001), fear of crime, subjective victimization risks, and increase perceived likelihood of bystander intervention (Vrij and Winkel, 1991). The improvement of public lighting is also being studied in light of environmental issues. The use of warm and uniform lights can ensure a sufficient level of feeling of safety and achieve energy savings (Saad et al., 2021). Benefits can also be expected from some luminaire types. For instance, street lighting with dynamic tracking can improve safety and recognition while using lower lighting levels (Wang et al., 2025).

Results concerning motivations, norms, and values (dimension 4) indicate that citizens consider public street lighting very important (Meneses et al., 2016). The vast majority of citizens support measures to reduce public lighting (Beaudet et al., 2022; Meneses et al., 2016). Citizens are open to changes in favor of more sustainable public lighting (Beaudet et al., 2022). Citizens may support changes in public lighting if they are intended to save energy and environmental purposes (Beaudet et al., 2022). This attitude and the impacts of ALAN influence support for the lighting policy (Bordel et al., 2025). More precisely, individuals who recognize the negative impacts of ALAN are more willing to accept reduction measures, whereas those who mainly perceive its positive impacts tend to be more reluctant (Bordel et al., 2025). The acceptability of lighting reduction policies also appears to be influenced by perceived safety and value-consistent information about the environmental benefits of reducing public lighting (Boomsma and Steg, 2014a,b). These levers are reflected in citizens' openness to certain changes, particularly with regard to lighting periods, e.g., between 1 a.m. and 5 a.m. (Beaudet et al., 2022). Also, any extension of public lighting beyond sunrise and sunset hours is perceived as unnecessary (Meneses et al., 2016). They are also open to changes in luminaire type. For example, LED and lamps with a high Scotopic/Photopic ratio are more accepted (de Oliveira Grando and Ghisi, 2021). Neutral white LEDs were also better accepted than yellow HPS lamps (Meneses et al., 2016). Findings regarding LED acceptance are nevertheless mixed. One study reported lower customer acceptance of LED solutions compared to other lighting options (Jayaratne et al., 2022). However, this openness to change faces several obstacles. Citizens express concerns and are less willing to accept changes to lighting for safety reasons than for reasons related to the physical aspects of light (Beaudet et al., 2022; Peña-García et al., 2024). Citizens reject solutions that compromise their safety (Meneses et al., 2016). Poorly lit environments with a higher risk of becoming trapped are perceived equally by men and women as less acceptable (Boomsma and Steg, 2014b). They also reject lighting solutions that interfere with their daily routines, such as radical changes to street lighting timetables. Finally, the higher the population density, the less willing residents are to accept lighting policies, such as turning off lights between 1 a.m. and 5 a.m. or between 11 p.m. and 6 a.m. (Beaudet et al., 2022).

For the fifth dimension, perceived organizational constraints, no article examines the perceived or organizational constraints related to artificial outdoor lighting.

Finally, the last dimension, behaviors and practices observed, revealed that during nighttime, lighting design affects the overall use of open public spaces, such as duration, frequency, activities, and individual spatial distribution (Rakonjac et al., 2022). Lighting characteristics also appear to play a role in space use. For instance, LED lamps improve spatial orientation (de Oliveira Grando and Ghisi, 2021). Visit frequency itself shapes mental states and environmental perception of lightscapes: higher visit frequency enhances perceptions of the spatial atmosphere but slightly reduces emotional and social evaluations of the lightscape (Huang et al., 2024). In turn, visit frequency is influenced by contextual factors: individuals are more willing to visit urban green spaces under artificial light than under moonlight (Zhao et al., 2023). Moreover, lighting influences walking time of pedestrians: individuals walk less than 30 min per week after dark (89.4%) (Park and Garcia, 2020). Yet, these lighting effects seem more related to its presence than to its characteristics. Indeed, the difference between the type of luminaire (fluorescent tube luminaires, commercial LEDs, and optimized LEDs) and the color rendering of warm white LEDs (Markvica et al., 2019; Rahm and Johansson, 2021) has no effect on pedestrian behavior. The presence of lighting also influences driver behavior. Significant differences are observed in driving errors, lapses, and risk perception between conditions with and without street lighting (Liu et al., 2021). These differences can be explained by the multiple factors influencing the speed choice. At the individual level, speed behavior is associated with gender, driving experience, frequency of nighttime driving each week and the average annual mileage (Liu et al., 2020). In addition, drivers' abilities to operate in low-light conditions plays a key role in their speed behavior (Liu et al., 2020). Under low-visibility conditions and without street lighting, technical driving skills influence speed behaviors, while the ability to perceive risks is reduced (Liu et al., 2020). These driving patterns very depending on speed limits. On a road with a 120 km/h speed limit, driving speed is largely influenced by driving's low-light abilities (Liu et al., 2020). The ability to perceive risk reduces driving speed on roads with speed limits of 80 and 120 km/h (Liu et al., 2020). The influence of risk perception on driving behavior is also more pronounced among specific groups. Elderly and more experienced drivers are less likely to adopt risky behaviors in low-light conditions (Liu et al., 2021). Contextual factors also influence drivers' speed choices. Drivers' speed preferences appear to remain consistent across different lighting conditions. Driving speed in a street-lit environment at night has a positive effect on speed selection in low-light conditions (Liu et al., 2020). Similarly, driving in a dimly lit environment is associated with speed behaviors in the absence of street lighting (Liu et al., 2020). Finally, changes in lighting type do not necessarily affect the driving experience. Presence-sensitive and conventional lighting provide similar levels of perceived pleasantness, uniformity and glare (Pihlajaniemi et al., 2023).

## 4 Discussion

The objective of this scoping review was to provide an overview of the literature on individuals' relationship with ALAN through the lens of psychosocial responses, rather than as a fully comprehensive account of all human-light interactions. More specifically, it aimed to (1) identify methodological approaches, (2) map the main dimensions studied, and (3) report findings concerning psychosocial responses with outdoor ALANs. The review identified 75 articles relevant to the research question. Based on the analysis of these articles, four main findings emerged: (1) the literature on outdoor ALAN is recent and geographically limited, (2) studies have a similar design but present a wide variety of ALAN operationalization and measures, which limits understanding of the actual psychosocial responses to light, (3) research on outdoor ALAN is dominated by a perceptual and emotional approach, while perceived constraints are never studied, and (4) theoretical frameworks, particularly in environmental psychology, are rarely used. All studies were analyzed using three complementary theoretical frameworks from environmental psychology (i.e., NAM, VBN, and CADM). This methodology provides a more comprehensive understanding of psychosocial responses to outdoor ALAN.

Several recent regulatory frameworks could explain this increased scientific interest, as light is now considered a pollutant in several regions of the world [e.g., the Dark Skies Protection Act in 2021–2022 in New York; Revised Municipal Environmental Protection Regulation in 2022 in Shanghai; Regulation (EU) 2020/852 in 2020 in Europe]. The intensification of research on psychosocial responses related to outdoor ALAN could also be linked to the global increase in energy costs (IEA, 2023), which has led to the implementation of policies aimed at reducing or optimizing nighttime lighting (e.g., EESL, 2020; U.S. DOE, 2019). Among the articles analyzed, there is also a concentration of studies in China, the United States, and European countries, i.e., highly urbanized countries where artificial lighting is significant. These highly industrialized countries face high levels of light pollution (Falchi et al., 2016; Lorenz, 2025), which explains why psychosocial responses to outdoor ALAN are more frequently studied in these countries. Similarly, most of the countries in which these studies are conducted are subject to variations in daylight, particularly during specific seasons (e.g., in Nordic countries, winter days are short and are characterized by limited daylight, Raza et al., 2024), which may influence dependence on outdoor ALAN and the different psychosocial responses individuals have to it (e.g., feelings of safety, comfort).

Analysis of the selected studies reveals a preference for quantitative, cross-sectional, and experimental methodologies, while qualitative, mixed-method, and longitudinal research remains less prevalent. Yet the use of complementary methods, particularly qualitative ones, could be useful in better understanding people's perceptions of their experiences (Lim, 2025). Longitudinal studies could also increase in-depth understanding of the dynamics of the phenomena being studied, such as changes in attitudes or behaviors (Caruana et al., 2015). The use of such methodologies therefore appears to be an opportunity for researchers to gain a better understanding of the processes at play (Creswell and Creswell, 2017; Vogl, 2023). The results of the review also highlight a wide variety of approaches to studying ALAN (i.e., manipulation, perception, or simulation of ALAN in real-world or laboratory settings). These different approaches are sometimes accompanied by objective, quantitative measurements of light using physical instruments, but this is not always the case. Furthermore, ALAN is defined differently across studies, meaning that not all studies focus on exactly the same subject. However, psychosocial responses (e.g., perception, safety, emotions) depend on how ALAN is studied (Cleary-Gaffney et al., 2022; van Rijswijk and Haans, 2018). This makes it difficult to compare results across different studies and to build a cumulative knowledge on ALAN.

This scoping review also reports significant heterogeneity in the measurement tools used in the selected studies. This finding has direct consequences for the field, as it limits the possibility of comparing and replicating studies (Bhattacherjee, 2012). The development of standardized tools and protocols, as well as interdisciplinary approaches, seems essential in order to promote reliability, transferability, and the possibility of applying the results to different contexts (NASEM, 2019; Pavic et al., 2025).

The results of this review reveal that research on ALAN is dominated by a perceptual and emotional approach (e.g., Burattini et al., 2025; Rahm and Johansson, 2021; Masullo et al., 2022) used to evaluate modifications in the physical parameters of outdoor ALAN (e.g., lighting levels, color temperature). More specifically, the results highlight a strong focus on perceptual and affective responses such as visibility, glare, comfort, and emotional reactions, often examined in relation to specific lighting configurations. Although these changes relate to the physical properties of lighting, their assessment is based primarily on subjective perceptions and affective responses that capture individuals' immediate experience of lighting conditions rather than the broader psychological processes underlying behavioral responses to lighting environments. This observation reflects a technical and utilitarian approach to lighting, which focuses primarily on optimizing physical properties, often giving less attention to individuals' understanding and opinions of ALAN (e.g., Davies and Smyth, 2018; Landvreugd et al., 2024). This also applies to other areas of engineering science. In the field of thermal comfort, for example, research is dominated by biophysical approaches (i.e., temperature, energy efficiency, Custódio et al., 2024). In the context of outdoor ALAN, the focus of research on perceptual and emotional aspects seems to lead to a still limited understanding of the psychosocial processes involved in responses to ALAN. These dimensions play a very limited role in the behavioral frameworks used in this review, which mainly explain individual behaviors through personal norms (Schwartz, 1977), environmental value orientations (Stern, 2000), and behavioral habits (Klöckner and Blöbaum, 2010). Thus, the perceptual and emotional assessments reported in the literature may reflect individuals' immediate experience in lighted environments, but they remain insufficient to provide a complete account of the normative and motivational processes underlying behaviors.

Conversely, perceived constraints have never been studied in any of the studies in this corpus. It is as if behaviors related to light (e.g., the willingness or unwillingness to adopt an extinction measure or to accept public lighting reduction policies) were studied in a decontextualized manner. This is particularly relevant given that acceptance or rejection of public lighting reduction policies appears to be one of the main behavioral outcomes examined in this scoping review (Beaudet et al., 2022; Boomsma and Steg, 2014a; Bordel et al., 2025; Meneses et al., 2016). Similarly, the CADM (Klöckner and Blöbaum, 2010) highlights the existing relationships between dimensions related to constraints, habitual processes, norms, intentions and behavior. Therefore, without taking this specific dimension into account, the results concerning the understanding of lighting policy decisions may be limited. However, several studies show that individuals take into account what they perceive as situational constraints (i.e., their own context) when assessing the difficulty of adopting pro-environmental behaviors (Attari et al., 2016; Fesenfeld, 2025). The lack of studies on this dimension constitutes a blind spot in the literature, as well as in the understanding and interpretation of current results. Future studies should incorporate measures of perceived constraints (e.g., contextual, social, or material) and their interactions with other key dimensions such as beliefs, emotions, and habits.

Analysis of these studies also reveals that none of the articles examined its results according to a theoretical analytical framework. This lack of theoretical foundation limits the relevance and consistency of the variables studied (Miyaguchi, 2022). In order to better understand the nature of psychosocial responses to outdoor ALAN, it seemed appropriate to analyse these variables using a framework based on theoretical models rooted in environmental psychology. This framework also highlighted that responses to ALAN does not depend solely on preferences or specific perceptions, but could be part of a coherent psychosocial system that can mobilize beliefs, expectations, and even emotions (e.g., Marshall et al., 2019; Williamson and Thulin, 2022). This framework therefore explains how individuals can construct meaning around lighting (Tian and Liu, 2022; Wittenberg et al., 2023). From this perspective, the assessment of a change concerning ALAN (e.g., reduction policy) cannot be understood by limiting oneself to isolated dimensions (e.g., safety, comfort, or environment), but depends on how they are articulated. Indeed, the results are sometimes ambivalent. This variability reflects the diversity of lighting parameters, environmental contexts, and individual characteristics examined across studies. In addition, the findings are highly fragmented across a wide range of specific outcomes (e.g., comfort, glare, visibility, preference), often depending on specific lighting parameters (e.g., illuminance, color temperature), as illustrated by divergent results (e.g., Davidovic et al., 2019b,a; Rahm and Johansson, 2021; Masullo et al., 2022).

For example, some studies included in this scoping review showed that individuals were in favor of lighting for reasons of safety, comfort, or modernity (e.g., Himschoot et al., 2024; Shahar et al., 2018; Zhao et al., 2023), while others report growing concern about light pollution and a possible desire to reduce ALAN (Falchi et al., 2016; Coogan et al., 2020). To interpret these contradictory results, it seems necessary to place them in the context of the relationship between personal norms (i.e., NAM), values (i.e., VBN), and routines rooted in nighttime practices (i.e., CADM). Consequently, for measures to be considered acceptable, each stakeholder would need to strike a balance between their preferences and aversions (e.g., Munroe, 2019), since failure to achieve, this balance could lead to a radical rejection of change (e.g., Carattini et al., 2018; Contzen et al., 2021). The framework proposed in this review highlights that the current literature focuses primarily on certain psychosocial dimensions in isolation rather than within validated theoretical frameworks that structure their theoretical relationships. It provides insight into the factors that must be taken into account for a measure to be perceived as legitimate, proportionate, and compatible with the everyday experience of the nighttime environment.

### 4.1 Strengths and limitations

This scoping review has several strengths. First, it was conducted with enhanced methodological quality by following the PRISMA-ScR recommendations (Tricco et al., 2018), by several researchers, with disagreements resolved by a third researcher. Similarly, a risk of bias analysis was performed (Peters et al., 2021), and an ICC was calculated to ensure transparency in this process (Koo and Li, 2016). Secondly, each included study was analyzed on the basis of a framework derived from theoretical models widely attested in the literature (NAM, Schwartz, 1977; VBN, Stern, 2000; CADM, Klöckner and Blöbaum, 2010). The framework used thus allows for a systematic and objective analysis, while illustrating how the studies address the issue of the social relationship with the object that is outdoor ALAN. It also offers the possibility of analyzing the findings through concepts from environmental psychology, even if the authors of the included studies did not explicitly consider them from this perspective.

Nevertheless, this work has several limitations. First, a systematic review or meta-analysis could not be conducted due to significant heterogeneity in objectives, measures, etc. Similarly, due to the emerging literature on human relationships with this specific object, a scoping review seemed to be the best option for identifying knowledge from this field of study, certain limitations inherent in the current literature, and future research perspectives (Munn et al., 2018; Peters et al., 2021). Second, this review focuses specifically on psychosocial responses to artificial light at night (ALAN) (e.g., perceptions, emotions, norms, and behaviors). As such, other dimensions of individuals' relationship with ALAN, such as health, wellbeing, or recreational and restorative experiences, remain less represented in this synthesis, which may limit the generalizability of the findings (Gaston et al., 2013; Wang et al., 2023). Third, the analytical framework derived from environmental psychology was applied *post hoc* to organize and interpret the findings, rather than to guide the selection of studies. While this approach allowed for a structured reading of a heterogeneous literature, it may also introduce interpretative constraints, as some results do not fully align with the predefined dimensions, reflecting the iterative and flexible nature of scoping review methodologies (Arksey and O'Malley, 2005; Levac et al., 2010). Fourth, although the synthesis aimed to be as comprehensive as possible, it remains primarily descriptive and does not constitute a fully integrative or meta-analytic analysis, which limits the ability to draw strong causal or general conclusions, as scoping reviews are primarily designed to map and characterize the literature rather than to provide quantitative or causal synthesis (Munn et al., 2018; Peters et al., 2020). Fifth, articles published in the literature generally present positive and significant results (Dwan et al., 2013), leading to biases in the conclusions and implications of research or applications highlighted by this scoping review. Sixth, despite a search strategy designed to be as comprehensive as possible, and aimed at maintaining a balance between specificity and sensitivity (Bramer et al., 2018), it is likely that some relevant terms or articles have been left out (Uttley et al., 2023). Similarly, the use of specific available databases may have led to the exclusion of some studies relevant to this topic (Schuller et al., 2025). In order to reduce this limitation, additional research was conducted in Google Scholar. Seventh, a large number of articles initially identified by the research question were excluded, reflecting the still incomplete nature of the literature on this topic (Rodrigo-Comino et al., 2023; Bordel et al., 2025). Eight, only articles published in English were selected, which may have led to the omission of articles addressing the research questions investigated (Pham et al., 2014). Finally, most of the included studies were conducted in similar geographical contexts (e.g., China and the United States), which may limit the generalisability of the results and conclusions to other cultural or geographical contexts (Charlson et al., 2021).

### 4.2 Implications for practice and for future research

This scoping review highlights several practical implications for researchers and policymakers involved in projects related to outdoor ALAN. However, several avenues for future studies can be identified. First, the geographical concentration of the work requires diversification of research at the cultural and territorial levels. Such research would provide a better understanding of how the environment, experiences, and the use of light influence individuals' behaviors and perceptions of ALAN (Coetzee et al., 2023). Indeed, these cultural and contextual aspects influence psychosocial responses with outdoor ALAN. For example, the perception of light pollution differs between urban and rural areas (Coogan et al., 2020). Cultural and contextual differences can therefore influence the acceptance of environmental projects (Renoth et al., 2023) and thus invite future research to conduct such studies in order to identify certain obstacles and levers inherent in these issues and projects. This scoping review also highlights the need to more clearly define the outcomes being assessed, to specify how to operationalize ALAN, and to better integrate objective measures with perceived experiences related to ALAN. This review also highlights the need for future studies to diversify the methodologies used, relying more on qualitative, mixed, or longitudinal approaches. Such methodologies enable an optimized understanding of perceptions and experiences and allow for the tracking of changes in certain dimensions investigated over time (Caruana et al., 2015; Kazdin, 2021; Lim, 2025; Vogl, 2023). This scoping review also reinforces the need for research to focus more specifically on understanding psychosocial dimensions that have been little studied in the literature (e.g., motivations, norms, values, and perceived constraints) as well as the relationships between these determinants. Finally, although there are many theoretical models in environmental psychology (e.g., VBN, Stern, 2000; CADM, Klöckner and Blöbaum, 2010) relevant to studying psychosocial responses of users and public lighting, research should nevertheless produce a theoretical framework specific to lighting. Such a framework would provide a better understanding of individuals' ambivalent psychosocial responses to artificial lighting while improving the reproducibility of studies. Similarly, there are currently a few specific scales for measuring connectedness with the night sky (e.g., Barnes and Passmore, 2024), but very few specific to individual's psychosocial responses to ALAN. Future studies should therefore draw on this type of work and the results of this scoping review to fill this gap.

This scoping review also reveals several practical implications for lighting reduction policies. While such reductions may appear necessary (e.g., biodiversity; reduction in energy costs), these reduction policies must be accompanied by consideration of the psychosocial implications related to outdoor ALAN. For example, the results of this review showed that even though citizens are in favor of lighting regulation for ecological reasons, they reject solutions that could affect their safety or daily lives (Meneses et al., 2016). Clearly, engaging in a discussion on reducing public lighting is not just a technical issue (Pérez Vega et al., 2021), as psychosocial issues (e.g., safety, well-being; Messa et al., 2025; Lanini-Maggi et al., 2024), environmental (e.g., pollution, biodiversity; Candolin, 2024; Hölker et al., 2021; Longcore, 2024) and political issues (e.g., acceptability, territorial justice; Boomsma and Steg, 2014b; Xiao et al., 2023). It is therefore through multidisciplinarity that issues ranging from the most technical to the most social can be reconciled (Maxwell et al., 2019). Some of the included studies also suggest that the acceptability of lighting reduction measures varies depending on the urban context. Higher population density is associated with lower acceptability of lighting policies (Beaudet et al., 2022). Citizens reluctant to reduce lighting primarily cite safety concerns (Beaudet et al., 2022). Conversely, in suburban areas, the dimming of streetlights appears to be more widely accepted (Beaudet et al., 2022). This effect is even more pronounced when information on environmental benefits is provided (Boomsma and Steg, 2014a). Furthermore, perceptions of light pollution also differ between urban and rural contexts (Coogan et al., 2020). These results suggest that lighting or lighting reduction strategies should therefore be tailored to the characteristics of each geographic area and should not be applied uniformly across the territory. Furthermore, citizens' values and attitudes are thought to play a role in the acceptability of reducing ALAN (Boomsma and Steg, 2014b; Beaudet et al., 2022). Public policies could therefore benefit from engaging in participatory approaches with the various stakeholders to jointly develop light reduction measures (Bonney et al., 2009; Nadarajah et al., 2024). Several types of solutions could be jointly developed or evaluated. For example, a certain level of lighting could be maintained along major pedestrian routes, while the intensity of lighting on some less-traveled streets would be reduced. Adaptive lighting systems that allow residents to adjust brightness levels (for example, via mobile apps or neighborhood-level control interfaces) could also be offered. Such solutions could also foster collective ownership of lighting decisions. For example, local outdoor ALAN management systems could allow residents to adjust lighting intensity (e.g., apps; connected boxes). This would also provide a better understanding of the motivations, norms, values, and perceived constraints associated with reducing outdoor ALAN. As a result, the issue of artificial lighting would no longer be addressed from a purely technical and utilitarian perspective, but rather from a psychosocial perspective aimed at collective appropriation of lighting through its recognition as a common good (Agrawal et al., 2023; Ostrom, 1990).

## Data Availability

The original contributions presented in the study are included in the article/Supplementary material, further inquiries can be directed to the corresponding author.
