# Supplementary material for: Psychosocial responses to outdoor artificial light at night (ALAN): a scoping review
Source: Front Psychol. 2026 May 29;17:1797689. doi: 10.3389/fpsyg.2026.1797689 (PMC13259720; doi:10.3389/fpsyg.2026.1797689)
Supplement: Supplementary file 1 [file Table_1.docx]

# Supplementary material S1. Search strategy

**Table S1.** Search results from the seven databases.

| Database /Date searched | EBSCO | | | | | | Web of Science | Google Scholar | Sum |
| --- | --- | --- | --- | --- | --- | --- | --- | --- | --- |
|  | Academy search premier | APA PsycInfo | Greenfile | Medline | Psychology and Behavioral Sciences Collection | Sum (EBSCO) |  |  |  |
| Number of articles found from the searches | 715 | 133 | 128 | 376 | 15 | 1367 | 1540 | 3 | 2910 |
| Number of duplicates |  | | | | | 372 | 57 | 0 | 429 |
| Total number minus duplicates |  | | | | | 995 | 1483 | 3 | 2481 |

**Figure S1**

*Research strategy used for developing the search equation (format adapted from the diagram of Moher et al., 2009)*

*
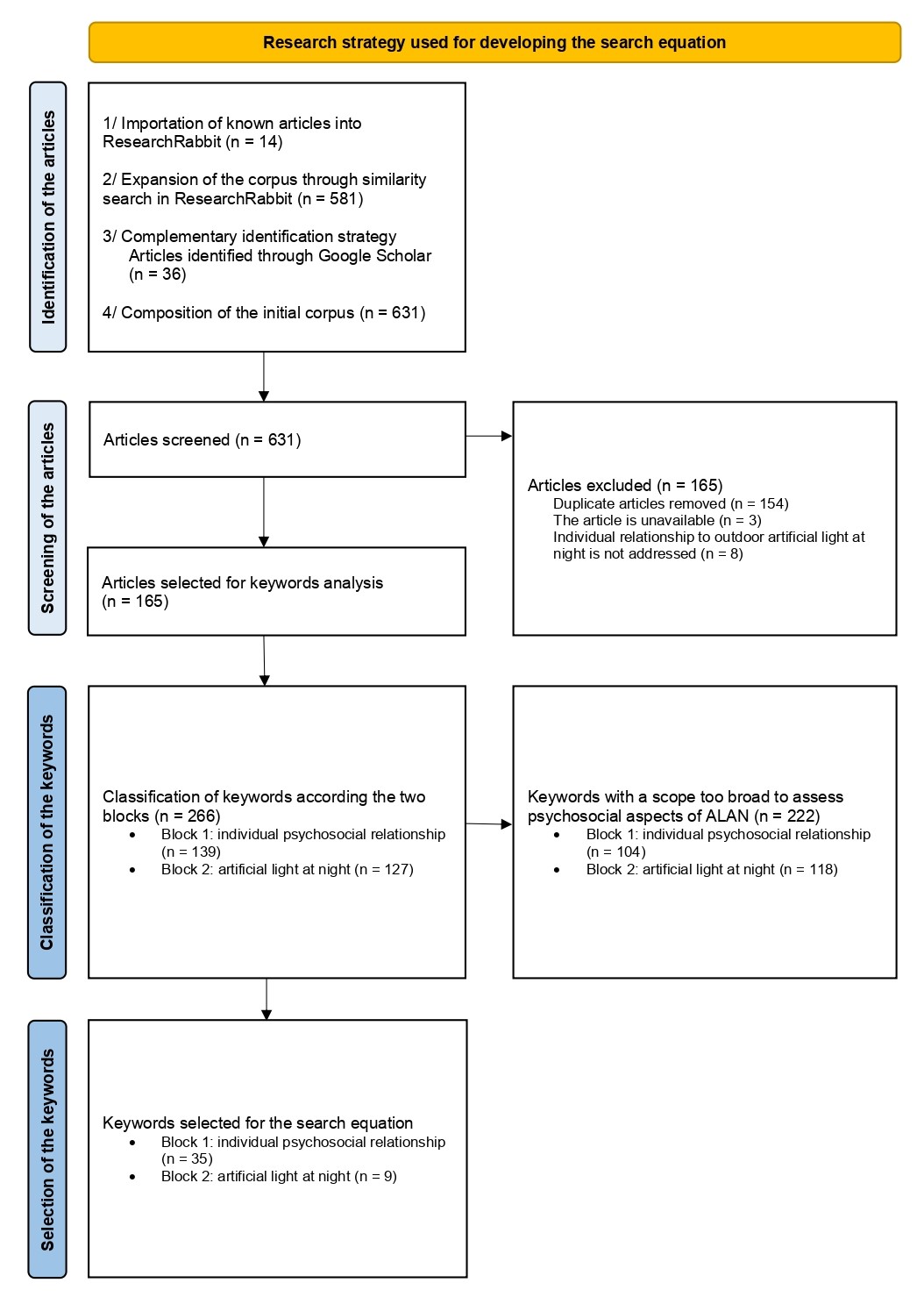
*
